# Correction: Gender and mental health of adolescents: A conceptual framework developed in a Delphi study

**DOI:** 10.1371/journal.pone.0346634

**Published:** 2026-04-08

**Authors:** The PLOS One Editors

## Notice of Republication

This article [1] was republished on March 13, 2026, to address an issue identified post-publication. An updated version of S1 Data is provided with this notice. Please download this article again to view the correct version.

The article’s Data Availability statement is updated to: All relevant data are within the paper and its Supporting Information files.

## Supporting information

S1 Data Data Delphi Study. (ZIP)
