# Supplementary material for: Gender and mental health of adolescents: A conceptual framework developed in a Delphi study
Source: PLoS One. 2025 Dec 15;20(12):e0318394. doi: 10.1371/journal.pone.0318394 (PMC12704890; doi:10.1371/journal.pone.0318394)
Supplement: S2 Table — (DOCX) [file pone.0318394.s002.docx]

**Supporting information S2 Table. Results of the proposed gender attitudes in the first Delphi round.**

| What gender norms can you think of that particularly influence adolescents? |
| --- |
| Girls |
| - Emotional - Type of toys: dolls - Norms to express oneself/ appearance/ aesthetics: colours (pink), type of clothes, wearing make-up, smile, being pretty - Quiet, not speaking up, silent, taking up less space - Being ostracized for being too sassy - Performing in school: underscoring in scientific/ mathematical subjects - Less physically active - Adhering to male gaze 🡪constantly under self or others surveillance - Being more aware of others’ than own needs, household responsibilities, caregiving for younger siblings - Quitting school to help mother with household chores - Choosing care-related occupations, e.g. nurse, teacher - Sexual debut/ activity, slut vs virgin, no sex before marriage, promiscuous if too much sex - Family expectations, e.g. child marriage, must give birth - Having taste - Being everything at once: effortlessly pretty, caring, successful, - Submissiveness - Limited to “safe” environments |
| Boys |
| - Choosing occupations that promise financial success - Suppressing emotions - Acting tough, even to one’s own disadvantage (drinking alcohol, smoking, engaging in physical fights, physical pain) - Teenage male circumcision - Constantly proving masculinity (athletic engagement, rejecting “gay” behaviours - Being muscular - Taking more responsibilities from outside the house - Allowed to venture out - Learning skills like riding a bicycle or swimming - Self-sacrifice - Physical over mental health - Taking risks - Working hard - Being logical - Type of toys, e.g. guns - Being macho, cool, hard, out-going, “class-clown”, not showing weaknesses, e.g. crying - Physically active - Having the final say, responsible for making decisions - Performing in school subjects: good in scientific, mathematical subjects - Romantic relationships: being sexually experiences, having many partners, partner at a certain age - Expectations about body shape, features, body weight - Norms around freedom/ parental monitoring (expectation of excessive freedom, being too old for instructions) - Socially awarded “assertiveness” - Expectation to be the breadwinner and taking financial/ material care for family - Toxic masculinity |
| Which gender norms do you think are of particular importance for the mental health of adolescents? Please write your ideas for gender norms and their possible impact for the mental health of adolescents into the text box. You can also repeat those you mentioned in the previous question. |
| Girls |
| - More emotional 🡪feelings not being taken seriously - Restrictions related to self-expression (toys - clothes) 🡪 may impair identity development and lead to them having to lead a double life - being raised with the idea that their first priority should be to get married 🡪could hinder/ clash with their ability to decide for themselves what self-actualization means - being raised not to speak up or vocalize their ideas or concerns 🡪affects their self-image and their self-confidence - Norms surrounding expected body shape, features and body weight - regarding appearance and aesthetics, body norms etc. - Being limited to safe environments 🡪 not able to take calculated risks when growing up - learning limited skills set 🡪 not able to contribute equally to household and outer responsibilities as an adult leading to perpetuating the gender roles further - sexual debut/sexual activity (slut v virgin) - sexuality norms, appropriate sexual behaviours - body image and appearance - for women that new sense of empowerment and "not giving a shit" often feels to me like a lot of repressed anger, trauma and sadness... seeming strong might actually be counter-productive to managing healthy boundaries and relationships - Getting to know their own identity without having role models, because often parents are still behaving according to old role models (binary, woman with children, man working and earning money) - Man is the leader can lead the sense of pride and a burden to men to show his strength. Also men can use violence to push disciplined toward women as subordinate to men. Woman is subordinated to men and weak must be protected by men which made women dependent and insecure. Women dress code which imposed by the dogma such as forced hijab - Non-appropriate behaviour (e.g. not adhering to certain roles/ stereotypes) of adolescent affects how the community views the whole family 🡪family support for adolescent may change 🡪mental health issues (feelings of loneliness, lack of support, not being able to share emotions) 🡪 leading to drug and alcohol use, becoming withdrawn - Stigma about sexuality 🡪leads to depression - Early pubertal development - girls developing secondary sexual characteristics like breast earlier than usual 🡪leads to emotional distress and depression - There is some evidence that "traditional" gender norms 🡪 are associated with decreased risk among women; it seems likely that this applies to adolescents, too. There is considerably less and inconsistent evidence for "internalizing" disorders (e.g., depression, anxiety), which tend to more prevalent among women. - sexualization of predominately cis-girls but also other genders 🡪 victims can feel reduced to looks, lower self-worth, use of abuse behaviour towards the victim ("she asked for it wearing that skirt") - unequal responsibilities and overburdening 🡪 frustration, stress, anxiety, depression |
| Boys |
| - being outgoing, physically active, a "class clown" (for boys) 🡪 overdiagnosis of ADHS in boys and potentially underdiagnosis of depression - not learning how to express different emotions 🡪expressing feelings as anger 🡪potential of violence - Restrictions related to self-expression (toys - clothes) 🡪 impair identity development and lead to them having to lead a double life - Norms surrounding expected body shape, features and body weight - Sexuality norms, appropriate sexual behaviours - Body image and appearance - for men I think it is hard to grapple with their loss of the breadwinner role, that now they need to "deliver" more, there is some resentment towards that which can lead to political radicalisation/isolation/rumination which can lead to anger, violence or depression - Getting to know their own identity without having role models, because often parents are still behaving according to old role models (binary, woman with children, man working and earning money) - Not admitting to weaknesses 🡪 not seeking help - Non-appropriate behaviour (e.g. not adhering to certain roles/ stereotypes) of adolescent affects how the community views the whole family 🡪family support for adolescent may change 🡪mental health issues (feelings of loneliness, lack of support, not being able to share emotions) 🡪 leading to drug and alcohol use, becoming withdrawn - Stigma about sexuality 🡪 leads to depression - There is some evidence that "traditional" gender norms are associated with greater risk of substance use (disorders) and other "externalizing" behaviors among men; it seems likely that this applies to adolescents, too - toxic masculinity 🡪 abusive behaviour |
