# Supplementary material for: Gender and mental health of adolescents: A conceptual framework developed in a Delphi study
Source: PLoS One. 2025 Dec 15;20(12):e0318394. doi: 10.1371/journal.pone.0318394 (PMC12704890; doi:10.1371/journal.pone.0318394)
Supplement: S3 Table — Full length. (DOCX) [file pone.0318394.s003.docx]

**Supporting information S3 Table. Consensus percentage of the quantitative questionnaire items of Delphi round 3 (n=9)**. Full length.

| Section | Questionnaire items | Consensus  % (n) |
| --- | --- | --- |
| Section A: Gender | | |
| Hypothesised causal influences starting from sex assigned at birth | Sex assigned at birth has an influence on gender attitudes | **Yes: 100 (9)** |
|  | Sex assigned at birth has an influence on gender roles | Yes: 67 (6) |
|  | Sex assigned at birth has an influence on competencies | Yes: 44 (4) |
|  | Sex assigned at birth has an influence on mental health | **Yes: 89 (8)** |
|  | Sex assigned at birth has an influence on gender identity | **Yes: 78 (7)** |
|  | Sex assigned at birth has an influence on gender norms (social environment) | Yes: 56 (5) |
| Hypothesised causal influences starting from gender identity | Gender identity has an influence on gender attitudes | **Yes: 78 (7)** |
|  | Gender identity has an influence on gender roles | **Yes: 89 (8)** |
|  | Gender identity has an influence on competencies | **Yes: 78 (7)** |
|  | Gender identity has an influence on mental health | **Yes: 89 (8)** |
|  | Gender identity has an influence on gender norms (social environment) | Yes: 44 (4) |
| Hypothesised causal influences starting from gender attitudes | Gender attitudes have an influence on gender roles | **Yes: 78 (7)** |
|  | Gender attitudes have an influence on competencies | **Yes: 78 (7)** |
|  | Gender attitudes have an influence on mental health | **Yes: 89 (8)** |
|  | Gender attitudes have an influence on gender identity | Yes: 33 (3) |
|  | Gender attitudes have an influence on gender norms (social environment) | Yes: 67 (6) |
| Hypothesised causal influences starting from gender roles | Gender roles have an influence on gender attitudes | Yes: 44 (4) |
|  | Gender roles have an influence on competencies | **Yes: 78 (7)** |
|  | Gender roles have an influence on mental health | **Yes: 89 (8)** |
|  | Gender roles have an influence on gender identity | Yes: 11 (1) |
|  | Gender roles have an influence on gender norms (social environment) | Yes: 33 (3) |
| Hypothesised causal influences starting from competencies | Competencies have an influence on gender attitudes | Yes: 33 (3) |
|  | Competencies have an influence on gender roles | Yes: 22 (2) |
|  | Competencies have an influence on mental health | **Yes: 89 (8)** |
|  | Competencies have an influence on gender identity | Yes: 0 (0) |
|  | Competencies have an influence on gender norms (social environment) | Yes: 56 (5) |
| Hypothesised causal influences starting from gender norms (social environment) | Gender norms have an influence on gender attitudes | **Yes: 89 (8)** |
|  | Gender norms have an influence on competencies | Yes: 67 (6) |
|  | Gender norms have an influence on mental health | **Yes: 89 (8)** |
|  | Gender norms have an influence on gender identity | Yes: 56 (5) |
|  | Gender norms have an influence on gender roles | **Yes: 100 (9)** |
| Gender roles | Agreement with operationalisation of gender roles: time spent on gender-typed activities | **Yes: 78 (7)** |
| Variables forming an intersectional lens | Relevance of the proposed social positions for adolescents | **Yes: 78 (7)** |
|  | Completeness of the proposed social positions for an intersectional perspective | Yes: 44 (4) |
| Section C: Social environment | | |
| Inclusion of actors most likely to influence gender attitudes | Family | **Yes: 100 (9)** |
|  | Peers | **Yes: 100 (9)** |
|  | School environment | **Yes: 100 (9)** |
|  | Sport club | Yes: 67 (6) |
|  | Faith club | Yes: 67 (6) |
|  | Hobby-based clubs | Yes: 56 (5) |
|  | Role models | Yes: 56 (5) |
|  | Celebrities | Yes: 67 (6) |
|  | Influencers | **Yes: 89 (8)** |
|  | Traditional leaders | Yes: 22 (2) |
|  | Media | Yes: 67 (6) |
|  | Social media | **Yes: 100 (9)** |
|  | Pornography | Yes: 44 (4) |
|  | Work environment | Yes: 22 (2) |
|  | Healthcare providers | Yes: 33 (3) |
|  | Law enforcement | Yes: 22 (2) |
|  | Political parties | Yes: 33 (3) |
|  | Civil Society Organisations | Yes: 33 (3) |
|  | Non-Profit Organisations | Yes: 33 (3) |
| Social environment level for only consented actor | Family - **Household** | **100 (9)** |
|  | Peers - **Community** | **100 (9)** |
|  | School environment - **Community**  - Political | 11 (1) |
|  | Influencers - **Digital** | **100 (8)** |
|  | Social media - Community  - **Digital** | **89 (8)** |
