# Supplementary material for: Gender and mental health of adolescents: A conceptual framework developed in a Delphi study
Source: PLoS One. 2025 Dec 15;20(12):e0318394. doi: 10.1371/journal.pone.0318394 (PMC12704890; doi:10.1371/journal.pone.0318394)
Supplement: S4 Table — (DOCX) [file pone.0318394.s004.docx]

**Supporting information S4 Table. Questionnaire Delphi survey round 2.**

| **Section: Overall** | |
| --- | --- |
| CP | **[Always display]**  Consent to participation / Consent to privacy policy |
| ID | **[Always display]**  Pseudonymization variable  To identify you over the three Delphi rounds, we introduced a 4-digit acronym. Please enter the first two letters of your mother's name plus the two last numbers of your year of birth. |
| **Section A: Gender** | |
| A1 | **[Always display, multiple choice possible]**  These gender concepts are included in the conceptual framework:   - Gender identity - Sex assigned at birth [Assigned sex is a label given at birth based on medical factors, such as hormones, chromosomes, and genitals. Most people are assigned male or female, which is registered on their birth certificate]   If any, which of those following gender concepts should also be included in the conceptual framework on gender (norms), the social environment and the mental health of adolescents? Please keep in mind that we can only include the most relevant aspects into the quantitative framework.  1: Sex/gender expression  2: Sex/gender relations  3: Sex/gender roles  4: Sexual orientation (sexuality)  99: No indication |
| A1-1-O | **[Always display]**  If you wish, please comment on your rating!  1: [free text box]  99: No indication |
| A1-2 | **[Always display]**  Are you aware of any (validated) instrument(s) that covers gender identity?  1: No  2: Yes |
| A1-3-O | **[Display if A1-2=2]**  Please tell us the name of the (validated) instrument(s).  1: [free text box]  99: No indication |
| A1-4-O | **[Display if A1-2=1]**  Please suggest possible questionnaire items that cover gender identity.  1: [free text box]  99: No indication |
| A1-5 | **[Always display]**  Are you aware of any (validated) instrument(s) that covers sex assigned at birth?  1: No  2: Yes |
| A1-6-O | **[Display if A1-5=2]**  Please tell us the name of the (validated) instrument(s).  1: [free text box]  99: No indication |
| A1-7-O | **[Display if A1-5=1]**  Please suggest possible questionnaire items that cover sex assigned at birth.  1: [free text box]  99: No indication |
| A1-8 | **[Display if A1=1]**  Are you aware of any (validated) instrument(s) that covers sex/gender expression?  1: No  2: Yes |
| A1-9-O | **[Display if A1-8=2]**  Please tell us the name of the (validated) instrument(s).  1: [free text box]  99: No indication |
| A1-10-O | **[Display if A1-8=1]**  Please suggest possible questionnaire items that cover sex/gender expression.  1: [free text box]  99: No indication |
| A1-11 | **[Display if A1=2]**  Are you aware of any (validated) instrument(s) that covers sex/gender relations?  1: No  2: Yes |
| A1-12-O | **[Display if A1-11=2]**  Please tell us the name of the (validated) instrument(s).  1: [free text box]  99: No indication |
| A1-13-O | **[Display if A1-11=1]**  Please suggest possible questionnaire items that cover sex/gender relations.  1: [free text box]  99: No indication |
| A1-14 | **[Display if A1=3]**  Are you aware of any (validated) instrument(s) that covers sex/gender roles?  1: No  2: Yes |
| A1-15-O | **[Display if A1-14=2]**  Please tell us the name of the (validated) instrument(s).  1: [free text box]  99: No indication |
| A1-16-O | **[Display if A1-14=1]**  Please suggest possible questionnaire items that cover sex/gender roles.  1: [free text box]  99: No indication |
| A1-17 | **[Display if A1=4]**  Are you aware of any (validated) instrument(s) that covers sexual orientation (sexuality)?  1: No  2: Yes |
| A1-18-O | **[Display if A1-17=2]**  Please tell us the name of the (validated) instrument(s).  1: [free text box]  99: No indication |
| A1-19-O | **[Display if A1-14=1]**  Please suggest possible questionnaire items that cover sexual orientation (sexuality).  1: [free text box]  99: No indication |
|  | |
| A2 | **[Always display, multiple choice possible]**  These gender approaches are reflected in the conceptual framework:   - Multidimensionality approach - Multilevel approach - Intersectionality approach - Gender power relations lens   If any, which of the following gender approaches should also be reflected in the conceptual framework? Please keep in mind that we can only include the most relevant aspects into the quantitative framework.  1: Gender continuum  2: Gender spectrum  3: Embodiment approach  4: Decolonial lens  99: No indication |
| A2-1-O | **[Always display]**  If you wish, please comment on your rating!  1: [free text box]  99: No indication |
| A2-2-O | **[Always display]**  The gender approaches will not be presented as their own categories in the conceptual framework. Instead, they will be reflected by the other categories (gender, gender norms, social environment levels, mental health) and the way these categories are represented in the framework.  For example: the multilevel approach is reflected in the different social environment levels that are relevant for adolescents and contain actors as carriers of gender norms.  How can the multidimensionality approach be reflected in the framework?  1: [free text box]  99: No indication |
| A2-3-O | **[Always display]**  How can the multilevel approach be reflected in the framework?  1: [free text box]  99: No indication |
| A2-4-O | **[Always display]**  How can the intersectionality approach be reflected in the framework?  1: [free text box]  99: No indication |
| A2-5-O | **[Always display]**  How can the power relations lens be reflected in the framework?  1: [free text box]  99: No indication |
| A2-6-O | **[Display if A2=1]**  How can the gender continuum be reflected in the framework?  1: [free text box]  99: No indication |
| A2-7-O | **[Display if A2=2]**  How can the gender spectrum be reflected in the framework?  1: [free text box]  99: No indication |
| A2-8-O | **[Display if A2=3]**  How can the embodiment approach be reflected in the framework?  1: [free text box]  99: No indication |
| A2-9-O | **[Display if A2=4]**  How can the decolonial lens be reflected in the framework?  1: [free text box]  99: No indication |
| A3 | **[Always display, multiple choice possible]**  Derived from all your proposed gender norms, we are suggesting categorizations for gender norms. We are suggesting two axes of categorizations.  1: This is our *first* suggestion for a categorization of gender norms (for boys and girls). It was derived from the suggestions in round 1 and covers the content-related areas where gender norms occur.   - Behaviour norms [Norms or expectations around how people of a certain gender (should) behave in a given situation] - Body and appearance norms [Norms or expectations regarding the appearance, aesthetics and body shape of people of a certain gender] - Sexual and relationship norms [Norms or expectations regarding acceptable attitudes or behaviours in relation of sexuality and relationships of people of a certain gender] - Performance norms [Norms or expectations regarding the success of people of a certain gender in certain subjects] - Education norms [Norms or expectations regarding the level of school attendance or attainment of people of a certain gender] - Mobility norms [Norms or expectations regarding the degree of mobility of a certain gender] - Career norms [Norms or expectations regarding the degree to which people of a certain gender (should) make a career and in what type of career]   2: This is our *second* suggestion for a categorization of gender norms. This suggestion adds descriptive [refer to what people of a certain gender are or what they do] and prescriptive norms [refer to what people of a certain gender should do] to the content-related areas of gender norms.   \|  \| Descriptive norms \| Prescriptive norms \| \| --- \| --- \| --- \| \| Behaviour norms \| E.g.:  Boys are stoic  Girls are emotional \| E.g.:  Boys should be strong  Girls should be empathic \| \| Body and appearance norms \|  \|  \| \| Sexual and relationship norms \|  \|  \| \| Performing norms \|  \|  \| \| Education norms \|  \|  \| \| Mobility norms \|  \|  \| \| Career norms \|  \|  \|   3: None of these suggestions  99: No indication |
| A3-1-O | **[Always display]**  If you wish, please comment on your rating!  1: [free text box]  99: No indication |
| A3-2-O | **[Always display]**  If you want, please propose another suggestion for a categorization of gender norms.  1: [free text box]  99: No indication |
| A3-3-O | **[Always display]**  Are you aware of any existing models/theories/frameworks that include any relevant categorization of gender norms? If so, please describe.  1: [free text box]  99: No indication |
|  | |
| A4-O | **[Always display]**  Is there something you would like to add that has not been addressed in this part or is there something you would like to comment on?  1: No  2: Yes [free text box]  99: No indication |
| **Section B: Mental health** | |
| B1 | **[Always display, multiple choice possible]**  These mental health outcomes are included in the conceptual framework:   - Mental, social and physical well-being - Depressiveness - Connectedness - Body image - Happiness - Risky behaviour   If any, which of the following mental health outcomes should also be included in the conceptual framework on gender (norms), the social environment and the mental health of adolescents? Please keep in mind that we can only include the most relevant aspects into the quantitative framework.  1: Self-efficacy  2: Self-control  3: Life purpose  4: Suicidal behaviour  5: Resilience  6: Substance misuse  7: Sense of coherence  99: No indication |
| B1-1 | **[Always display]**  If you wish, please comment on your rating!  1: [free text box]  99: No indication |
| B1-2 | **[Always display]**  Are you aware of any (validated) instrument(s) that covers mental, social and physical well-being?  1: No  2: Yes |
| B1-3-O | **[Display if B1-2=2]**  Please tell us the name of the (validated) instrument(s).  1: [free text box]  99: No indication |
| B1-4-O | **[Display if B1-2=1]**  Please suggest possible questionnaire items that cover mental, social and physical well-being.  1: [free text box]  99: No indication |
| B1-5 | **[Always display]**  Are you aware of any (validated) instrument(s) that covers depressiveness?  1: No  2: Yes |
| B1-6-O | **[Display if B1-5=2]**  Please tell us the name of the (validated) instrument(s).  1: [free text box]  99: No indication |
| B1-7-O | **[Display if B1-5=1]**  Please suggest possible questionnaire items that cover depressiveness.  1: [free text box]  99: No indication |
| B1-8 | **[Always display]**  Are you aware of any (validated) instrument(s) that covers connectedness?  1: No  2: Yes |
| B1-9-O | **[Display if B1-8=2]**  Please tell us the name of the (validated) instrument(s).  1: [free text box]  99: No indication |
| B1-10-O | **[Display if B1-8=1]**  Please suggest possible questionnaire items that cover connectedness.  1: [free text box]  99: No indication |
| B1-11 | **[Always display]**  Are you aware of any (validated) instrument(s) that covers body image?  1: No  2: Yes |
| B1-12-O | **[Display if B1-11=2]**  Please tell us the name of the (validated) instrument(s).  1: [free text box]  99: No indication |
| B1-13-O | **[Display if B1-11=1]**  Please suggest possible questionnaire items that cover body image.  1: [free text box]  99: No indication |
| B1-14 | **[Always display]**  Are you aware of any (validated) instrument(s) that covers happiness?  1: No  2: Yes |
| B1-15-O | **[Display if B1-14=2]**  Please tell us the name of the (validated) instrument(s).  1: [free text box]  99: No indication |
| B1-16-O | **[Display if B1-14=1]**  Please suggest possible questionnaire items that cover happiness.  1: [free text box]  99: No indication |
| B1-17 | **[Always display]**  Are you aware of any (validated) instrument(s) that covers risky behaviour?  1: No  2: Yes |
| B1-18-O | **[Display if B1-14=2]**  Please tell us the name of the (validated) instrument(s).  1: [free text box]  99: No indication |
| B1-19-O | **[Display if B1-14=1]**  Please suggest possible questionnaire items that cover risky behaviour.  1: [free text box]  99: No indication |
| B1-20 | **[Display if B1=1]**  Are you aware of any (validated) instrument(s) that covers self-efficacy?  1: No  2: Yes |
| B1-21-O | **[Display if B1-20=2]**  Please tell us the name of the (validated) instrument(s).  1: [free text box]  99: No indication |
| B1-22-O | **[Display if B1-20=1]**  Please suggest possible questionnaire items that cover self-efficacy.  1: [free text box]  99: No indication |
| B1-23 | **[Display if B1=2]**  Are you aware of any (validated) instrument(s) that covers self-control?  1: No  2: Yes |
| B1-24-O | **[Display if B1-23=2]**  Please tell us the name of the (validated) instrument(s).  1: [free text box]  99: No indication |
| B1-25-O | **[Display if B1-23=1]**  Please suggest possible questionnaire items that cover self-control.  1: [free text box]  99: No indication |
| B1-26 | **[Display if B1=3]**  Are you aware of any (validated) instrument(s) that covers life purpose?  1: No  2: Yes |
| B1-27-O | **[Display if B1-26=2]**  Please tell us the name of the (validated) instrument(s).  1: [free text box]  99: No indication |
| B1-28-O | **[Display if B1-26=1]**  Please suggest possible questionnaire items that cover life purpose.  1: [free text box]  99: No indication |
| B1-29 | **[Display if B1=4]**  Are you aware of any (validated) instrument(s) that covers suicidal behaviour?  1: No  2: Yes |
| B1-30-O | **[Display if B1-29=2]**  Please tell us the name of the (validated) instrument(s).  1: [free text box]  99: No indication |
| B1-31-O | **[Display if B1-29=1]**  Please suggest possible questionnaire items that cover suicidal behaviour.  1: [free text box]  99: No indication |
| B1-32 | **[Display if B1=5]**  Are you aware of any (validated) instrument(s) that covers resilience?  1: No  2: Yes |
| B1-33-O | **[Display if B1-32=2]**  Please tell us the name of the (validated) instrument(s).  1: [free text box]  99: No indication |
| B1-34-O | **[Display if B1-32=1]**  Please suggest possible questionnaire items that cover resilience.  1: [free text box]  99: No indication |
| B1-35 | **[Display if B1=6]**  Are you aware of any (validated) instrument(s) that covers substance misuse?  1: No  2: Yes |
| B1-36-O | **[Display if B1-35=2]**  Please tell us the name of the (validated) instrument(s).  1: [free text box]  99: No indication |
| B1-37-O | **[Display if B1-35=1]**  Please suggest possible questionnaire items that cover substance misuse.  1: [free text box]  99: No indication |
| B1-38 | **[Display if B1=7]**  Are you aware of any (validated) instrument(s) that covers sense of coherence?  1: No  2: Yes |
| B1-39-O | **[Display if B1-38=2]**  Please tell us the name of the (validated) instrument(s).  1: [free text box]  99: No indication |
| B1-40-O | **[Display if B1-38=1]**  Please suggest possible questionnaire items that cover sense of coherence.  1: [free text box]  99: No indication |
|  | |
| B2 | **[Always display]**  Is there something you would like to add that has not been addressed in this part or is there something else you would like to comment on?  1: [free text box]  99: No indication |
| **Section C: Social environment** | |
| C1 | **[Always display, multiple choice possible]**  The following illustration of social environment levels was updated based on the suggestions in the previous Delphi round.  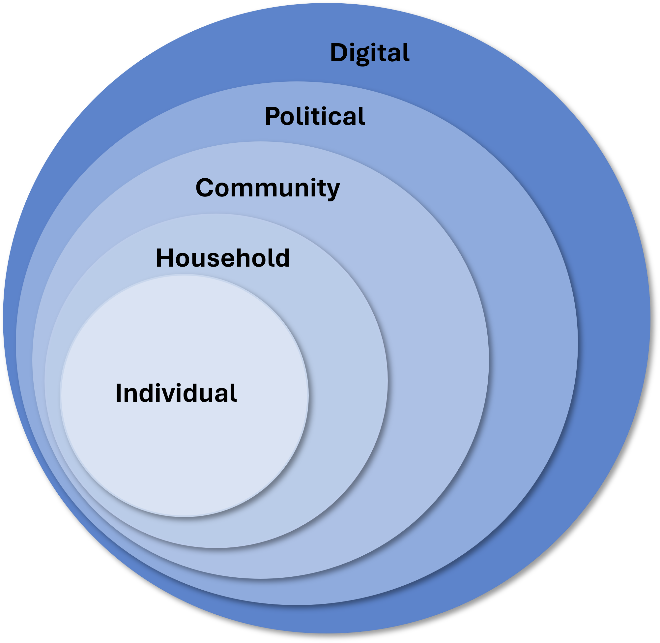  Please note that the social environment levels are not operationalised themselves. The levels serve as “buckets” for the actors who are carriers of gender norms. Please keep in mind that we can only include the most relevant aspects into the quantitative framework.  **[Always display]**  Do you think this illustration is appropriate for the conceptual framework?  1: No  2: Yes |
| C1-1-O | **[Display if C1=1]**  Please make another suggestion for the social environment levels.  1: [free text box]  99: No indication |
| C1-2-O | **[Always display]**  If you wish, please comment on your rating!  1: [free text box]  99: No indication |
|  | |
| C2 | **[Always display, multiple choice possible]**  The following competencies for adolescents are included in the conceptual framework:   - Coping skills - Agency - Interpersonal relationship skills - Critical reflection skills - Mental health literacy - Respect and empathy for others   If any, which of those competencies for adolescents should also be included in the conceptual framework on gender (norms), the social environment and the mental health of adolescents? Please keep in mind that we can only include the most relevant aspects into the quantitative framework.  1: Media literacy  2: Self-awareness  3: Life literacy [on career, future etc.]  4: Self-efficacy  5: Spirituality  6: Assertive skills  7: Navigation  99: No indication |
| C2-1-O | **[Always display]**  If you wish, please comment on your rating!  1: [free text box]  99: No indication |
| C2-2 | **[Always display]**  Are you aware of any (validated) instrument(s) that covers coping skills?  1: No  2: Yes |
| C2-3-O | **[Display if C2-1=2]**  Please tell us the name of the (validated) instrument(s).  1: [free text box]  99: No indication |
| C2-4-O | **[Display if C2-1=1]**  Please suggest possible questionnaire items that cover coping skills.  1: [free text box]  99: No indication |
| C2-5 | **[Always display]**  Are you aware of any (validated) instrument(s) that covers agency?  1: No  2: Yes |
| C2-6-O | **[Display if C2-4=2]**  Please tell us the name of the (validated) instrument(s).  1: [free text box]  99: No indication |
| C2-7-O | **[Display if C2-4=1]**  Please suggest possible questionnaire items that cover agency.  1: [free text box]  99: No indication |
| C2-8 | **[Always display]**  Are you aware of any (validated) instrument(s) that covers interpersonal relationship skills?  1: No  2: Yes |
| C2-9-O | **[Display if C2-7=2]**  Please tell us the name of the (validated) instrument(s).  1: [free text box]  99: No indication |
| C2-10-O | **[Display if C2-7=1]**  Please suggest possible questionnaire items that cover interpersonal relationship skills.  1: [free text box]  99: No indication |
| C2-11 | **[Always display]**  Are you aware of any (validated) instrument(s) that covers critical reflection skills?  1: No  2: Yes |
| C2-12-O | **[Display if C2-10=2]**  Please tell us the name of the (validated) instrument(s).  1: [free text box]  99: No indication |
| C2-13-O | **[Display if C2-10=1]**  Please suggest possible questionnaire items that cover critical reflection skills.  1: [free text box]  99: No indication |
| C2-14 | **[Always display]**  Are you aware of any (validated) instrument(s) that covers mental health literacy?  1: No  2: Yes |
| C2-15-O | **[Display if C2-13=2]**  Please tell us the name of the (validated) instrument(s).  1: [free text box]  99: No indication |
| C2-16-O | **[Display if C2-13=1]**  Please suggest possible questionnaire items that cover mental health literacy.  1: [free text box]  99: No indication |
| C2-17 | **[Always display]**  Are you aware of any (validated) instrument(s) that covers respect and empathy for others?  1: No  2: Yes |
| C2-18-O | **[Display if C2-16=2]**  Please tell us the name of the (validated) instrument(s).  1: [free text box]  99: No indication |
| C2-19-O | **[Display if C2-16=1]**  Please suggest possible questionnaire items that cover respect and empathy for others.  1: [free text box]  99: No indication |
| C2-20 | **[Display if C2=1]**  Are you aware of any (validated) instrument(s) that covers media literacy?  1: No  2: Yes |
| C2-21-O | **[Display if C2-19=2]**  Please tell us the name of the (validated) instrument(s).  1: [free text box]  99: No indication |
| C2-22-O | **[Display if C2-19=1]**  Please suggest possible questionnaire items that cover media literacy.  1: [free text box]  99: No indication |
| C2-23 | **[Display if C2=2]**  Are you aware of any (validated) instrument(s) that covers self-awareness?  1: No  2: Yes |
| C2-24-O | **[Display if C2-22=2]**  Please tell us the name of the (validated) instrument(s).  1: [free text box]  99: No indication |
| C2-25-O | **[Display if C2-22=1]**  Please suggest possible questionnaire items that cover self-awareness.  1: [free text box]  99: No indication |
| C2-26 | **[Display if C2=3]**  Are you aware of any (validated) instrument(s) that covers life literacy?  1: No  2: Yes |
| C2-27-O | **[Display if C2-25=2]**  Please tell us the name of the (validated) instrument(s).  1: [free text box]  99: No indication |
| C2-28-O | **[Display if C2-25=1]**  Please suggest possible questionnaire items that cover life literacy.  1: [free text box]  99: No indication |
| C2-29 | **[Display if C2=4]**  Are you aware of any (validated) instrument(s) that covers self-efficacy?  1: No  2: Yes |
| C2-30-O | **[Display if C2-28=2]**  Please tell us the name of the (validated) instrument(s).  1: [free text box]  99: No indication |
| C2-31-O | **[Display if C2-28=1]**  Please suggest possible questionnaire items that cover self-efficacy.  1: [free text box]  99: No indication |
| C2-32 | **[Display if C2=5]**  Are you aware of any (validated) instrument(s) that covers spirituality?  1: No  2: Yes |
| C2-33-O | **[Display if C2-31=2]**  Please tell us the name of the (validated) instrument(s).  1: [free text box]  99: No indication |
| C2-34-O | **[Display if C2-31=1]**  Please suggest possible questionnaire items that cover spirituality.  1: [free text box]  99: No indication |
| C2-35 | **[Display if C2=6]**  Are you aware of any (validated) instrument(s) that covers assertive skills?  1: No  2: Yes |
| C2-36-O | **[Display if C2-34=2]**  Please tell us the name of the (validated) instrument(s).  1: [free text box]  99: No indication |
| C2-37-O | **[Display if C2-34=1]**  Please suggest possible questionnaire items that cover assertive skills.  1: [free text box]  99: No indication |
| C2-38 | **[Display if C2=7]**  Are you aware of any (validated) instrument(s) that covers navigation?  1: No  2: Yes |
| C2-39-O | **[Display if C2-37=2]**  Please tell us the name of the (validated) instrument(s).  1: [free text box]  99: No indication |
| C2-40-O | **[Display if C2-37=1]**  Please suggest possible questionnaire items that cover navigation.  1: [free text box]  99: No indication |
| **Section: Overall** | |
| C3 | Is there something you would like to add that has not been addressed in this part or is there something else you would like to comment on?  1: [free text box]  99: No indication |
| QE | Is there anything else you would like to share with us?  1: [free text box]  99: No indication |
