# Supplementary material for: Gender and mental health of adolescents: A conceptual framework developed in a Delphi study
Source: PLoS One. 2025 Dec 15;20(12):e0318394. doi: 10.1371/journal.pone.0318394 (PMC12704890; doi:10.1371/journal.pone.0318394)
Supplement: S5 Table — (DOCX) [file pone.0318394.s005.docx]

**Supporting information S5 Table. Questionnaire Delphi survey round 3.**

| Section: Overall | |
| --- | --- |
| CP | **[Always display]**  Consent to participation / Consent to privacy policy |
| ID | **[Always display]**  Pseudonymization variable:  To identify you over the three Delphi rounds, we introduced a 4-digit acronym. Please enter the first two letters of your mother's name plus the two last numbers of your year of birth. |
|  | |
|  | **[Always display]**  These are the constructs for the conceptual framework that are based on the results of Delphi round 2.  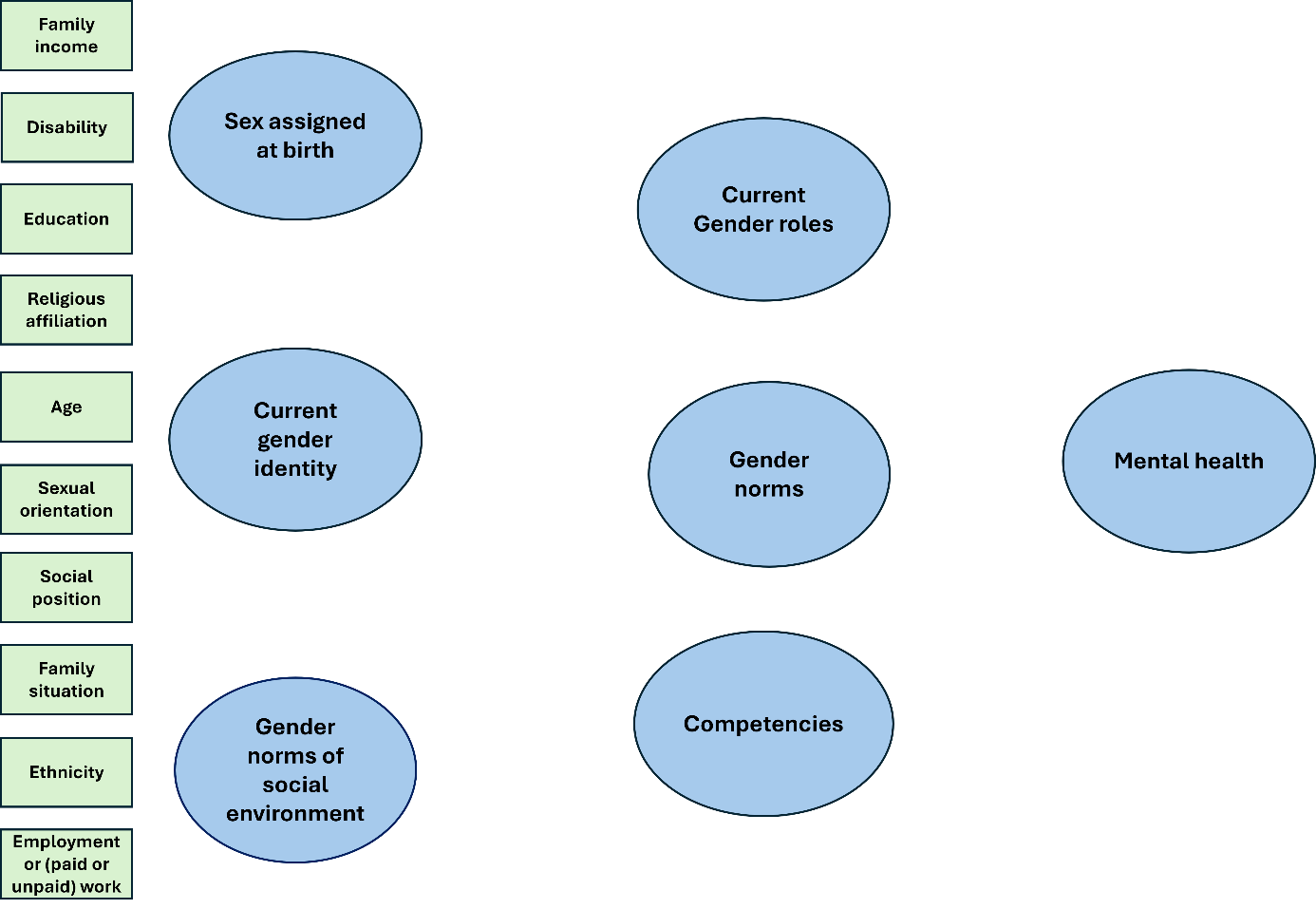  This initial conceptual framework includes the constructs that reached consensus in the first two Delphi rounds: **Gender norms of social environment, sex assigned at birth, gender identity, (individual) gender norms, competencies, gender roles and mental health**.   - **Gender norms of social environment** are measured by gender norms of (actors of) the household level; gender norms of (actors of) the community level; gender norms of (actors of) the political level and gender norms of (actors of) the digital level. - **(Individual) gender norms** are measured by behaviour norms; performance norms; body & appearance norms; education norms; sexual & relationship norms; career norms and mobility norms. - **Competencies** are measured by coping skills; agency skills; respect & empathy; mental health literacy; critical reflection skills and interpersonal relationship skills. - **Mental health**, depending on the research interest, is measured either by mental, social or physical well-being; depressiveness; connectedness; body image; resilience; happiness or risky behaviour.   We further integrated several **gender approaches** either directly or indirectly.   - We integrated the **multilevel approach** by integrating several social environment levels. - Additionally, we reflected the **multidimensionality approach** by developing sex and gender concepts with different dimensions (sex assigned at birth, gender identity, gender roles). - We reflected the **intersectionality approach** and the **gender power relations lens** by integrating multiple social positions in an intersectional perspective for the population group of adolescents that include aspects of power relations and processes of discrimination and touch upon the micro level but also reflect multiple interlocking systems of privilege and oppression at the macro level. |
| State-O | **[Always display]**  If you wish, please comment on the statements above!  1: [free text box]  99: No indication |
| Sex | **[Always display, multiple choice possible]**  We are now looking for assumptions about causal relationships (one event causes an effect of an other event, also referred to as cause-and-effect relationship) between these constructs for possible quantitative analyses.  Starting from **sex assigned at birth**, with which other construct(s) (in blue) would you assume a causal relationship?  1: Sex assigned at birth has an influence on individual gender norms  2: Sex assigned at birth has an influence on gender roles  3: Sex assigned at birth has an influence on competencies  4: Sex assigned at birth has an influence on mental health  5: Sex assigned at birth has an influence on gender identity  6: Sex assigned at birth has an influence on gender norms of social environment |
| Gi | **[Always display, multiple choice possible]**  Starting from **current gender identity**, with which other construct(s) (in blue) would you assume a causal relationship?  1: Gender identity has an influence on Individual gender norms  2: Gender identity has an influence on gender roles  3: Gender identity has an influence on competencies  4: Gender identity has an influence on mental health  5: Gender identity has an influence on gender norms of social environment |
| Env | **[Always display, multiple choice possible]**  Starting from **gender norms of social environment**, with which other construct(s) (in blue) would you assume a causal relationship?  1: Gender norms of social environment have an influence on individual gender norms  2: Gender norms of social environment have an influence on gender roles  3: Gender norms of social environment have an influence on competencies  4: Gender norms of social environment have an influence on mental health  5: Gender norms of social environment have an influence on gender identity |
| Gn | **[Always display, multiple choice possible]**  Starting from **individual** **gender norms**, with which other construct(s) (in blue) would you assume a causal relationship?  1: Gender norms have an influence on gender roles  2: Gender norms have an influence on competencies  3: Gender norms have an influence on mental health  4: Gender norms have an influence on gender identity  5: Gender norms have an influence on gender norms of social environment |
| Gr | **[Always display, multiple choice possible]**  Starting from **gender roles**, with which other construct(s) (in blue) would you assume a causal relationship?  1: Gender roles have an influence on gender norms  2: Gender roles have an influence on competencies  3: Gender roles have an influence on mental health  4: Gender roles have an influence on gender identity  5: Gender roles have an influence on gender norms of social environment |
| Comp | **[Always display, multiple choice possible]**  Starting from **competencies**, with which other construct(s) (in blue) would you assume a causal relationship?  1: Competencies have an influence on gender norms  2: Competencies have an influence on gender roles  3: Competencies have an influence on mental health  4: Competencies have an influence on gender identity  5: Competencies have an influence on gender norms of social environment |
| Comment-Causal-O | **[Always display]**  If you wish, please comment on your ratings!  1: [free text box]  99: No indication |
| **Section A: Gender** | |
| Oper-Gr | **[Always display]**  In the last Delphi round, there was a consensus on including gender roles in the conceptual framework. We propose operationalising gender roles with “time spent on gender-typed activities” (e.g. domestic chores for household, work in family business/income generating activities, care for others).  Do you find this proposition adequate to operationalise gender roles of adolescents?  1: Yes  2: No  99: No indication |
| Oper-Gr-Alternative | **[Display if Oper-Gr =2]**  Please suggest another operationalisation for gender roles.  1: [free text box]  99: No indication |
| Intersec-Relevance | **[Always display]**  Please take a look at the suggested social positions forming an intersectional perspective (in light green on the left of the proposed conceptual framework). Do you find the proposed social positions relevant for adolescents?  1: Yes  2: No  99: No indication |
| Intersec-Rel-Alternative | **[Display if Intersec-Rel =2]**  Please make another suggestion for the social position variables.  1: [free text box]  99: No indication |
| Intersec-Compl | Do you find the proposed social positions complete for an intersectional perspective?  1: Yes  2: No  99: No indication |
| Intersec-Compl-Alternative | **[Display if Intersec-Compl =2]**  Please make another suggestion for the social position variables.  1: [free text box]  99: No indication |
| **Section C: Social environment** | |
| Actors | **[Always display, multiple choice possible]**  In the last Delphi round, we concluded on the social environment levels (individual, household, community, political, digital). Please select the actors who are most likely to influence adolescents’ gender norms. We integrated your suggestions from the first Delphi round.  1: Family  2: Peers/Friends  3: School environment [e.g. teachers, classmates etc.]  4: Sport group  5: Faith group  6: Clubs (hobby-based groups)  7: Role models  8: Celebrities  9: Influencers  10: Traditional leaders  11: Media [e.g. television, newspapers, movies]  12: Social Media [e.g. Tik-tok, Instagram]  13: Pornography  14: Workplace  15: Healthcare providers  16: Political Parties 17: Law enforcement  18: Civil Society  19: Non-Profit Organisations |
| Fam | **[Display if Actors=1]**  Please assign the actor **family** to the social environment level in which it plays the greatest role.  1: Household level  2: Community level  3: Political (includes institutional) level  4: Digital level |
| Peer | **[Display if Actors =2]**  Please assign the actor **peers/friends** to the social environment level in which it plays the greatest role.  1: Household level  2: Community level  3: Political (includes institutional) level  4: Digital level |
| School | **[Display if Actors =3]**  Please assign the actor **school environment** to the social environment level in which it plays the greatest role.  1: Household level  2: Community level  3: Political (includes institutional) level  4: Digital level |
| Sport | **[Display if Actors =4]**  Please assign the actor **sport group** to the social environment level in which it plays the greatest role.  1: Household level  2: Community level  3: Political (includes institutional) level  4: Digital level |
| Faith | **[Display if Actors =5]**  Please assign the actor **faith group** to the social environment level in which it plays the greatest role.  1: Household level  2: Community level  3: Political (includes institutional) level  4: Digital level |
| Club | **[Display if Actors =6]**  Please assign the actor **hobby-based clubs** to the social environment level in which it plays the greatest role.  1: Household level  2: Community level  3: Political (includes institutional) level  4: Digital level |
| Role | **[Display if Actors =7]**  Please assign the actor **role models** to the social environment level in which it plays the greatest role.  1: Household level  2: Community level  3: Political (includes institutional) level  4: Digital level |
| Celebrities | **[Display if Actors =8]**  Please assign the actor **celebrities** to the social environment level in which it plays the greatest role.  1: Household level  2: Community level  3: Political (includes institutional) level  4: Digital level |
| Influencers | **[Display if Actors =9]**  Please assign the actor **influencers** to the social environment level in which it plays the greatest role.  1: Household level  2: Community level  3: Political (includes institutional) level  4: Digital level |
| Leader | **[Display if Actors =10]**  Please assign the actor **traditional leaders** to the social environment level in which it plays the greatest role.  1: Household level  2: Community level  3: Political (includes institutional) level  4: Digital level |
| Media | **[Display if Actors =11]**  Please assign the actor **media** to the social environment level in which it plays the greatest role.  1: Household level  2: Community level  3: Political (includes institutional) level  4: Digital level |
| Socialmedia | **[Display if Actors =12]**  Please assign the actor **social media** to the social environment level in which it plays the greatest role.  1: Household level  2: Community level  3: Political (includes institutional) level  4: Digital level |
| Porno | **[Display if Actors =13]**  Please assign the actor **pornography** to the social environment level in which it plays the greatest role.  1: Household level  2: Community level  3: Political (includes institutional) level  4: Digital level |
| Work | **[Display if Actors =14]**  Please assign the actor **workplace** to the social environment level in which it plays the greatest role.  1: Household level  2: Community level  3: Political (includes institutional) level  4: Digital level |
| Healthcare | **[Display if Actors =15]**  Please assign the actor **healthcare providers** to the social environment level in which it plays the greatest role.  1: Household level  2: Community level  3: Political (includes institutional) level  4: Digital level |
| PolParties | **[Display if Actors =16]**  Please assign the actor **political parties** to the social environment level in which it plays the greatest role.  1: Household level  2: Community level  3: Political (includes institutional) level  4: Digital level |
| Law | **[Display if Actors =17]**  Please assign the actor **law enforcement** to the social environment level in which it plays the greatest role.  1: Household level  2: Community level  3: Political (includes institutional) level  4: Digital level |
| Civil | **[Display if Actors =18]**  Please assign the actor **Civil Society** to the social environment level in which it plays the greatest role.  1: Household level  2: Community level  3: Political (includes institutional) level  4: Digital level |
| NGO | **[Display if Actors =19]**  Please assign the actor **Non-Profit Organisations** to the social environment level in which it plays the greatest role.  1: Household level  2: Community level  3: Political (includes institutional) level  4: Digital level |
| Comment-Envlevel | **[Always display]**  If you wish, please comment on your ratings!  1: [free text box]  99: No indication |
| Ameliorate-Framework | **[Always display]**  Is there anything you would like to mention or ameliorate concerning the conceptual framework at this stage?  1: [free text box]  99: No indication |
| **Section: Overall** | |
| QE | **[Always display]**  Looking back at the three Delphi rounds. Is there anything you would like to share with us?  1: [free text box]  99: No indication |
