# Supplementary material for: Gender and mental health of adolescents: A conceptual framework developed in a Delphi study
Source: PLoS One. 2025 Dec 15;20(12):e0318394. doi: 10.1371/journal.pone.0318394 (PMC12704890; doi:10.1371/journal.pone.0318394)
Supplement: Attachment — Submitted filename: Rebuttal letter_peer review_v3.docx [file pone.0318394.s009.docx]

**Rebuttal letter**

Dear Editorial Office and Reviewers,

Thank you for the opportunity to revise this manuscript. You will find our point-by-point responses to your revision requests below.

Kind regards,

The team

# Editor / Journal requirements:

When submitting your revision, we need you to address these additional requirements.

1. Please ensure that your manuscript meets PLOS ONE's style requirements, including those for file naming. The PLOS ONE style templates can be found at https://journals.plos.org/plosone/s/file?id=wjVg/PLOSOne_formatting_sample_main_body.pdf and <https://journals.plos.org/plosone/s/file?id=ba62/PLOSOne_formatting_sample_title_authors_affiliations.pdf>

We have gone through the style requirements and have made a couple of changes.

2. Your ethics statement should only appear in the Methods section of your manuscript. If your ethics statement is written in any section besides the Methods, please delete it from any other section.

We have verified that the ethics statement only appears in the ‘ethical considerations’ subsection of the methods section and that the file names adhere to the formatting requirements.

3. We note that you have indicated that there are restrictions to data sharing for this study. For studies involving human research participant data or other sensitive data, we encourage authors to share de-identified or anonymized data. However, when data cannot be publicly shared for ethical reasons, we allow authors to make their data sets available upon request. For information on unacceptable data access restrictions, please see <http://journals.plos.org/plosone/s/data-availability#loc-unacceptable-data-access-restrictions>.

Before we proceed with your manuscript, please address the following prompts:

a) If there are ethical or legal restrictions on sharing a de-identified data set, please explain them in detail (e.g., data contain potentially identifying or sensitive patient information, data are owned by a third-party organization, etc.) and who has imposed them (e.g., a Research Ethics Committee or Institutional Review Board, etc.). Please also provide contact information for a data access committee, ethics committee, or other institutional body to which data requests may be sent.

b) If there are no restrictions, please upload the minimal anonymized data set necessary to replicate your study findings to a stable, public repository and provide us with the relevant URLs, DOIs, or accession numbers. Please see http://www.bmj.com/content/340/bmj.c181.long for guidelines on how to de-identify and prepare clinical data for publication. For a list of recommended repositories, please see https://journals.plos.org/plosone/s/recommended-repositories. You also have the option of uploading the data as Supporting Information files, but we would recommend depositing data directly to a data repository if possible.

Please update your Data Availability statement in the submission form accordingly.

There are no restrictions. We have made our anonymised datasets available in the Supporting Information files.

4. In the online submission form, you indicated that all relevant data are within the manuscript and its Supporting Information files. We have removed personal and sensitive data due to the small sample size and the anonymous nature of the Delphi study. These data are available on reasonable request from the corresponding author.

All PLOS journals now require all data underlying the findings described in their manuscript to be freely available to other researchers, either 1. In a public repository, 2. Within the manuscript itself, or 3. Uploaded as supplementary information.

This policy applies to all data except where public deposition would breach compliance with the protocol approved by your research ethics board. If your data cannot be made publicly available for ethical or legal reasons (e.g., public availability would compromise patient privacy), please explain your reasons on resubmission and your exemption request will be escalated for approval.

We have updated our data availability statement: “All relevant data are within the manuscript and its Supporting Information files.”

5. We note that the grant information you provided in the ‘Funding Information’ and ‘Financial Disclosure’ sections do not match.

When you resubmit, please ensure that you provide the correct grant numbers for the awards you received for your study in the ‘Funding Information’ section.

We have updated and harmonised the funding information and financial disclosure: This paper is part of a PhD and was thereby funded within the scope of the Junior Research Group GendEpi (Gender Epidemiology) within the Department of Epidemiology & International Public Health, School of Public Health, Bielefeld University. The second funding phase of the Junior Research Group was funded by Bielefeld University [grant number not applicable, to CM]. The funders had no role in study design, data collection and analysis, decision to publish, or preparation of the manuscript.

6. Thank you for stating the following financial disclosure:

This paper is part of a PhD and was thereby funded within the scope of the Junior Research Group GendEpi (Gender Epidemiology) within the Department of Epidemiology & International Public Health, School of Public Health, Bielefeld University.

Please state what role the funders took in the study. If the funders had no role, please state: "The funders had no role in study design, data collection and analysis, decision to publish, or preparation of the manuscript."

If this statement is not correct you must amend it as needed.

Please include this amended Role of Funder statement in your cover letter; we will change the online submission form on your behalf.

We have added this sentence to our funding information and financial disclosure (see above).

7. Please include captions for your Supporting Information files at the end of your manuscript, and update any in-text citations to match accordingly. Please see our Supporting Information guidelines for more information: http://journals.plos.org/plosone/s/supporting-information.

8. We note that there is identifying data in the Supporting Information file <Ethikantrag Genehmigung_EUB-2023-111-S.pdf, Submission PLOS ONE Paper 2.zip>. Due to the inclusion of these potentially identifying data, we have removed this file from your file inventory. Prior to sharing human research participant data, authors should consult with an ethics committee to ensure data are shared in accordance with participant consent and all applicable local laws.

Data sharing should never compromise participant privacy. It is therefore not appropriate to publicly share personally identifiable data on human research participants. The following are examples of data that should not be shared:

-Name, initials, physical address

-Ages more specific than whole numbers

-Internet protocol (IP) address

-Specific dates (birth dates, death dates, examination dates, etc.)

-Contact information such as phone number or email address

-Location data

-ID numbers that seem specific (long numbers, include initials, titled “Hospital ID”) rather than random (small numbers in numerical order)

Data that are not directly identifying may also be inappropriate to share, as in combination they can become identifying. For example, data collected from a small group of participants, vulnerable populations, or private groups should not be shared if they involve indirect identifiers (such as sex, ethnicity, location, etc.) that may risk the identification of study participants.

Additional guidance on preparing raw data for publication can be found in our Data Policy (https://journals.plos.org/plosone/s/data-availability#loc-human-research-participant-data-and-other-sensitive-data) and in the following article: http://www.bmj.com/content/340/bmj.c181.long.

Please remove or anonymize all personal information (<specific identifying information in file to be removed>), ensure that the data shared are in accordance with participant consent, and re-upload a fully anonymized data set. Please note that spreadsheet columns with personal information must be removed and not hidden as all hidden columns will appear in the published file.

Thank you, we have anonymised all data.

9. If the reviewer comments include a recommendation to cite specific previously published works, please review and evaluate these publications to determine whether they are relevant and should be cited. There is no requirement to cite these works unless the editor has indicated otherwise.

# Response to Reviewers

## Reviewer 1

The research paper titled "Gender and Mental Health of Adolescents: A Conceptual Framework Developed in a Delphi Study" by Anita Alaze and colleagues explores the intersection of gender norms and mental health during adolescence. It aims to address the lack of a theoretical framework that conceptualizes the relationship between gender norms, attitudes, and mental health outcomes.

Key Highlights:

Objective: The study develops a conceptual framework to illustrate the interplay of key constructs in the gendered pathways of adolescent mental health using the Delphi technique2.

Methodology:

An international panel of 21 experts participated in three rounds of surveys.

The study identified core constructs, operationalized them, and hypothesized their relationships.

A 70% consensus threshold was used.

Findings:

Core constructs include gender norms, sex assigned at birth, gender attitudes, roles, competencies, and identity.

The framework incorporates intersectional, multidimensional, and multilevel approaches, along with a power relations lens.

Four social environment levels—household, community, political, and digital—were identified2.

Conclusion: The framework links six key constructs, four gender approaches, and four social environment levels to adolescent mental health. Future research is encouraged to validate and apply these relationships. This paper provides a valuable foundation for understanding the gendered pathways to mental health in adolescence.

Thank you very much for your positive feedback.

The practical implications of this study's findings can significantly influence multiple domains, especially in shaping policies, programs, and interventions aimed at improving adolescent mental health. Here are some key applications:

Mental Health Programs:

The framework provides a structure for designing mental health interventions tailored to address the gendered experiences of adolescents.

By recognizing the impact of gender norms and roles, practitioners can develop targeted therapies or workshops for adolescents struggling with gender-related mental health challenges.

Education Policies:

Schools can integrate the conceptual framework into their curricula to promote gender-inclusive environments.

Educators can create awareness among students about how gender attitudes and norms affect mental health.

Community Engagement:

Community-based programs can use this framework to advocate for cultural changes and challenge harmful gender norms impacting adolescents' mental health.

Digital Platforms:

The study highlights the influence of digital environments, suggesting opportunities to use online tools for mental health education and support.

Social media campaigns can be designed to promote healthier gender attitudes and provide resources for mental health care.

Policy Development:

Policymakers can apply the framework when drafting legislation focused on adolescent mental health, ensuring gender is a central consideration.

This could include funding for gender-sensitive mental health services and training for practitioners.

Intersectional Approaches:

The findings encourage the use of intersectionality in addressing mental health, helping practitioners and researchers to cater to diverse gender identities and socio-economic contexts.

By implementing these strategies, the conceptual framework developed by Anita Alaze's study offers transformative opportunities to advance adolescent well-being and mental health equity.

Thank you for highlighting these implications for (theory) and practice. We have added some of your suggestions to the last paragraph of the discussion section: “Potential implications for theory and practice following further validation of the framework include an intersectional perspective in designing more tailored interventions, workshops, policies, educational or community-based programs related to adolescents’ gender and/or mental health or community gender norms.”

Applying the findings from Anita Alaze's study may present several challenges due to the complexity of factors involved. Here are some key potential obstacles:

Cultural Resistance:

Deeply ingrained gender norms may face resistance from communities or institutions reluctant to change. Advocacy efforts may be slow to gain acceptance in certain cultural contexts.

Implementation Across Diverse Contexts:

Adolescents' experiences with gender and mental health vary widely based on culture, socio-economic background, and personal identity. Adapting the framework to meet these diverse needs could be complex.

Interdisciplinary Coordination:

Effectively addressing gendered pathways in mental health requires collaboration between educators, policymakers, healthcare providers, and community leaders. Aligning these sectors may be logistically challenging.

Resource Limitations:

Implementing gender-sensitive programs and interventions demands funding, trained professionals, and infrastructure, which may not be readily available in all regions.

Intersectionality Challenges:

The framework emphasizes intersectionality, but addressing multiple overlapping identities (e.g., gender, socioeconomic status, ethnicity) requires nuanced approaches that may not be well-established yet.

Digital Risks:

While the digital space offers opportunities for outreach, it also poses risks like cyberbullying, misinformation, and exposure to harmful content, which can undermine the benefits of mental health interventions.

Measurement and Validation:

The framework needs empirical testing and validation in real-world settings, which can be time-consuming and methodologically challenging.

Stigma and Awareness:

Persistent stigma around mental health and gender issues may deter adolescents from seeking help or participating in programs.

Overcoming these challenges will require concerted efforts to tailor interventions, promote cultural sensitivity, and secure necessary resources. It’s a reminder that progress in areas like mental health and gender equity is gradual but profoundly impactful.

The research paper concludes with the presentation of a conceptual framework that highlights the intricate relationship between gender norms, attitudes, and adolescent mental health. By identifying core constructs and employing a multidimensional, intersectional approach, the study provides a foundational tool for understanding the gendered pathways to mental health outcomes.

Key Takeaways from the Conclusion:

Framework Validation: The study underscores the importance of empirically testing and validating the proposed framework in diverse contexts to enhance its applicability.

Call for Action: It advocates for researchers, policymakers, educators, and mental health practitioners to incorporate gender-sensitive approaches in their work.

Future Directions: It encourages further exploration and refinement of the identified constructs and relationships to better support adolescent mental health globally.

Impactful Change: The framework sets the stage for challenging harmful gender norms and creating environments that foster equity and mental well-being.

The conclusion of this project highlights both its theoretical contributions and its potential for real-world impact, paving the way for meaningful interventions in adolescent mental health.

Thank you for underscoring the importance of our work. To reduce the obstacle of implementation across different contexts, we have highlighted that the operationalisations of the constructs are flexible. They should be adapted to the specific sociocultural context rather than adapting the conceptual framework to all potential sociocultural contexts (also see responses to reviewer 3). We agree that the adaptation of this conceptual framework to a specific sociocultural context is complex, but in our view, it is a necessary complexity to account for the fluid nature of gender.

We have recently submitted a manuscript in which we validated the conceptual framework among Ethiopian adolescents. This can serve as a methodological example for those aiming to validate this framework in other sociocultural settings.

## Reviewer 2

Dear authors,

Your contribution to knowledge is commendable. However, there is need to clearly raise a research question to guide your inquiry.

We have included our research question, which reads as follows: “To address this gap, our research question for this study is as follows: What are key constructs in the gendered pathways of health, from gender norms to adolescent’s mental health, and how do they interplay? Therefore, we aim to develop a conceptual framework that illustrates the interplay of key constructs in the gendered pathways of health, from gender norms to ado-lescent’s mental health.”

Although, it was later identified that your sample size is 20 experts. It will be fine if you can have a separate subheading for population and sample for clarity.

The first subheading of the results’ section is “Delphi Sample”. However, we agree that the sample size is only mentioned at the very end of this subchapter. We have now included the sample size in the first sentence of this section: “The median age of the initial 21 Delphi experts was 39 years (…)”

Lastly, there is need for you to mention the implications of your findings for theory and practice.

Thank you for this comment, which was also noted by another reviewer. We have added implications for theory and practice to the last paragraph of the discussion section: “Potential implications for theory and practice following further validation of the framework include an intersectional perspective in designing more tailored interventions, workshops, policies, educational or community-based programs related to adolescents’ gender and/or mental health or community gender norms. The conceptual framework could also guide variable selection for secondary data analysis within this research area.”

## Reviewer 3

While the objective of producing a theoretical framework for an understudied area is clear, several methodological and substantive concerns limit the paper's current contribution and warrant careful reconsideration. The framework organizes constructs across individual, relational, community, and societal levels, highlighting intersectionality and the complexity of gendered pathways - this is a strength of the paper, as is the synthesis attempt (to consolidate fragmented literature and expert opinion into one framework provides a starting point for discussion and hypothesis generation). However, there are methodological concerns.

First, there is a mismatch between method and subject matter. For adolescent gender norms (including links to mental health), a substantial empirical literature exists - including large-scale survey and qualitative studies (e.g., GEAS (which the authors mention), GEM scale, several publications in the Lancet of global gender norms analyses) - which could and should have informed framework development. By relying almost exclusively on expert opinion - and without adolescent participation - the study risks producing constructs that are unanchored from lived experiences.

Second, simplifying context-specific insights into standardized constructs. This is particularly problematic given evidence that gender norms vary across cultural contexts, age groups, and social environments. The authors do reference GEAS and GAGE as existing work but does not engage with their key findings. For example, GEAS demonstrates that adolescents' lived experiences reveal site-specific variations in gender norms, with age and gender-specific differences and culturally contingent social hierarchies. This contradicts the assumption that standardized, expert-defined constructs can fully capture gendered experiences. GAGE provides a conceptual framework that does not capture sex assigned at birth or gender identity (that the authors acknowledge), and its constructs are empirically grounded rather than purely theoretical. The authors claim that their framework is "more related to theoretical aspects" which ignores the importance of empirical validation and the context-specific variations documented in these studies (as well as others). By not fully incorporating these empirical insights, the Delphi framework risks representing expert assumption rather than adolescent realities.

The methodological references in the paper (e.g., Niederberger & Spranger, Jorm etc) outline proper Delphi procedures but simultaneously highlight that Delphi provides one of the lowest levels of evidence and should only be applied where higher-quality empirical methods are infeasible. Given the extensive literature on adolescent gender norms, the choice of Delphi is methodologically questionable and should be further explained. The framework's utility for guiding interventions or measurement development depends entirely on future validation. However, empirical studies suggest that starting with adolescent voices would likely yield substantially different constructs, reducing the framework's preliminary credibility.

Thank you very much for highlighting these important aspects. We will discuss each aspect in the following.

We have motivated the use of the Delphi study both in the preceding publication (Alaze, Finne, et al., 2025) and in this manuscript. We used the Delphi procedure and its consensus-generating methodology in order to identify the most relevant constructs necessary for this research question. Non-Delphi qualitative interviews and focus groups cannot generate consensus in this way. Quantitative analyses were not applicable to this research question, as our aim was to identify the most relevant constructs from the indeed extensive, inconsistent and sometimes conflicting scientific literature, and to then derive a conceptual framework. In our opinion, there was no study design or research method that is considered higher-level evidence that we could have used to disentangle the scientific literature and to employ the findings in a conceptual framework. Our procedure goes beyond of what a literature review could do. With the Delphi study, we have combined empirical evidence with expert knowledge that is not publicly available for synthesis. Given the conceptual inconsistencies in gender research, these perspectives are important because they are theory or practice based but not always taken into account.

My suggested improvements are: 1) Engage existing empirical literature, beyond GEAS and GAGE, and include key studies such as Weber et al, 2019 (Lancet), Kagesten et al, 2016 (mixed-methods review), and various GEM scale applications provide quantitative and qualitative evidence of age, gender, and context-specific variations that could have informed construct selection.

We have reviewed an extensive amount of literature prior to developing the Delphi study. In a previously published paper, we explain how we have developed the first round of the Delphi study based on the empirical literature (Alaze, Finne, et al., 2025). For instance, we have cited Kagesten et al., (2016) in the manuscript. In cognitive interviews and group discussions, we asked experts in gender, psychology, sociology, and adolescence whether we had missed important theoretical aspects from the literature. Due to this prior publication, to which we refer in this manuscript, we have prioritised the description of the three Delphi rounds in the current publication.

We have engaged with the findings of the GAGE project in the steps following the framework development. We have i) analysed the GAGE gender norms scale and its factor structure in the following publication (Alaze, Grosser, et al., 2025), and we have ii) just submitted another manuscript in which we empirically validate the conceptual framework in Ethiopian adolescents using secondary data analysis (Structural Equation Model).

2) Ground constructs in adolescent experience: incorporating youth perspectives either through mixed-methods consultation or secondary data analysis would enhance the conceptual validity of the framework (and/or include the reason as to why this was not able to be done/could not be done, rather than it being just a "limitation").

We agree that the perspectives of adolescents are imperative when conducting research about adolescents. We also support the idea to conduct a secondary data analysis as a means to include adolescent voices, as can be seen from our recently submitted manuscript validating the conceptual framework using GAGE data from Ethiopian adolescents. However, during the development phase of the conceptual framework, we were unable to integrate the adolescent perspectives, apart from involving a few early career professionals and experts on adolescence in the development of the questionnaire/framework. For this reason, we had originally planned to first develop the conceptual framework, which would then serve as a guide for selecting variables for secondary data analysis, and then to validate the framework and incorporate adolescents’ realities using GAGE data. A next step would be to conduct a study that recruits adolescents in Low Income Countries and to incorporate its findings into the conceptual framework. We have explained this limitation more extensively in the respective sections:

- We have added a paragraph at the beginning of the section strengths & limitations: “A significant limitation is that, apart from the inclusion of a few early career professionals in the Delphi study and an expert on adolescence during the development of the first Delphi round, we have not yet incorporated adolescents’ perspectives. As this carries the risk that the perspectives or needs of adolescents are not yet fully met, it is an important next step to validate the framework among adolescents using secondary data analysis or to complement and further expand the framework through qualitative interviews with adolescents.”
- We have also highlighted this in the last sentence of the conclusion: “Secondary data analysis could be particularly useful for validating the conceptual framework and integrating the lived realities of adolescents, but qualitative interviews, particularly with adolescents, could also complement and further expand the framework.”

3) Clarify theoretical vs. empirical positioning: Explicitly framing the framework as a hypothesis-generating exercise rather than an authoritative depiction of gendered pathways would set accurate expectations for subsequent research.

4) Contextual sensitivity: The framework should highlight that constructs may manifest differently across cultures and developmental stages (i.e., clarify why/how this would be flexible and adaptable as mentioned in the paper).

We did not have the intention to provide standardised constructs in the conceptual framework nor to assume that such standardised constructs can fully capture gendered experiences, as indicated by the statement: “Gender attitudes are subject to change, and are closely connected to culture (18), which is why we were reluctant to propose specific gender attitudes and proposed domains instead. Although this makes our framework less specific, it offers scope for flexible operationalisation”. The proposed gender norms operationalisation was intended to demonstrate possible key areas in which gender norms prevail rather than proposing a standardised operationalisation or specific questionnaire items/scales. In every empirical analysis, the operationalisation of gender norms (and of the other constructs) should be adapted to the specific sociocultural context (or research question). Our aim was therefore to demonstrate which gender dimensions are most relevant for our research objective.

To clarify the hypothesis-generating nature of the framework and to make the flexibility in the operationalisation of constructs more explicit, we have revised the manuscript in several places:

- We have adapted part of the conclusion, which now reads: “We believe that this framework has a hypothesis-generating nature and can be a starting point for more theory-driven research in this field. Our framework is more nuanced and adaptable than the few other frameworks on this topic and can shed light on more concrete gendered pathways to mental health. It is intended to be adapted to the specific sociocultural context and should be further refined and validated based on sociocultural contexts, new knowledge and different research methods.”
- We have similarly changed the first paragraph of the discussion section, which now reads: “The conceptual framework has a hypothesis-generating nature and is a first step in approaching the gendered pathways of mental health for the age group of adolescents and should therefore be understood as a flexible tool that can be adapted according to the specific sociocultural context and research objectives.”
- We have also changed an additional part of the discussion section, which now reads “Gender attitudes are subject to change, and are closely connected to culture (18), which is why we were reluctant to propose specific gender attitudes and proposed domains instead. Although this makes our framework less specific, it offers scope for flexible operationalisation. This means that the operationalisation of the constructs can be adapted, such as to the sociocultural context, data availability, or developmental stages of adolescents.”

Overall, the theoretical ambition is clear, and the Delphi method is correctly applied procedurally, but the framework is fundamentally limited by its lack of engagement with empirical evidence and adolescent perspectives. I recommend major revision, with particular attention to integrating existing empirical studies, explicitly addressing context specificity, and clarifying the framework's provisional, hypothesis-generating nature. This paper can provide great value as a starting point for theoretical discussion, but it must be revised to incorporate empirical grounding and adolescent perspectives. Without this, the framework risks codifying expert assumptions rather than capturing the lived realities of adolescence, and what the evidence base already says, which limits its relevance for research and intervention in the field of gender and mental health.

References

Alaze, A., Finne, E., Razum, O., & Miani, C. (2025). A questionnaire for a conceptual framework and interdisciplinary public health research using the Delphi technique-development and validation. *Frontiers in Public Health*, *13*, 1436569. https://doi.org/10.3389/fpubh.2025.1436569

Alaze, A., Grosser, J., Razum, O., & Miani, C. (2025). How does a recent gender norms scale perform? Exploratory factor analyses among adolescents in Ethiopia and Bangladesh. *PLOS Global Public Health*, *5*(10), e0005190. https://doi.org/10.1371/journal.pgph.0005190
