# Supplementary material for: Correction: Gender and mental health of adolescents: A conceptual framework developed in a Delphi study
Source: PLoS One. 2026 Apr 8;21(4):e0346634. doi: 10.1371/journal.pone.0346634 (PMC13061248; doi:10.1371/journal.pone.0346634)
Supplement: S1 Data — (ZIP) [file pone.0346634.s001.zip › Limesurvey_Questionnaire Delphi round 1.pdf]

LimeSurvey

Thank you very much for agreeing to participate in this Delphi study! You received this invitation for the survey because you are an expert in gender (norms), the social environment and the mental health of adolescents working either in research, policy, or development, services, and implementation.

With this low-barrier questionnaire, we want to involve you in the development of a conceptual framework on gender and gender norms, the social environment, and the mental health of adolescents to theoretically link these three aspects. We want to integrate research and practice-driven perspectives, and a diversity of points of view.

This questionnaire contains four sections. Section A is on gender (norms), section B on the mental health and section C on the social environment of adolescents. Ultimately, section D is about your person.

How to fill out this questionnaire?

Your thoughts/ opinions on various theories, models or frameworks will be inquired. You might not be an expert on all topics, however, to combine these three constructs, it is necessary to gain new knowledge on the relations between these topics. Therefore, please try to fill out all sections from your perspective but always keep in mind that the questions are asked for and related to all three topics in this framework. There is no right or wrong to the questions so please feel free to fill out the questions according to your opinion or knowledge. You may feel like all proposed aspects are somehow important. The goal of this questionnaire is to reduce the aspects to the most relevant ones to ultimately develop the conceptual framework which can be used in quantitative research. If you do not know some terms, you can click on the green question mark to get more information. Please note that these are examples of a definition and that other meanings of the term are also possible.

--

Declaration of participation

Your participation in this study is voluntary. Personal identifying characteristics (email address and other demographic data) are only available to the project team who is obliged to maintain confidentiality and only shares the data in such a way that no individual can be identified. The participating experts stay anonymous in the Delphi Survey.

For more information on the declaration of participation, please click on the term privacy policy or legal notice at the end of this page.

LimeSurvey

Section A: Pseudonymization

A1. **To identify the participants over the three Delphy rounds, we would like to introduce a 4-digit acronym. Please enter the first two letters of your mother's name plus the two last numbers of your year of birth.**

E. g. Emma born in 1961 = EM61

Section B: Section A: Gender

Sex/gender definitions:

We define sex as follows:

Sex refers to a set of *biological attributes*. It is primarily associated with physical and physiological features including chromosomes, gene expression, hormone levels and function, and reproductive/sexual anatomy. Sex is *usually categorized as female or male but there is variation* in the biological attributes that comprise sex and how those attributes are expressed.

We define gender as follows:

“Gender refers to the socially constructed roles, behaviours, expressions and identities of girls, women, boys, men, and gender diverse people. It *influences how people perceive themselves and each other, how they act and interact, and the distribution of power and resources* in society. Gender identity is not confined to a binary (girl/woman, boy/man) nor is it static; it exists along a continuum and can change over time. There is considerable diversity in how individuals and groups understand, experience and express gender through the roles they take on, the expectations placed on them, relations with others and the complex ways that gender is institutionalized in society”.

Although there is a distinct definition for the terms, sex/gender should not be considered separately because they interact with each other.

Section C: Section A: Gender

C1. **In addition to *gender identity* and *sex assigned at birth*, which of those other aspects of common gender concepts, theories or approaches do you think are most essential for the social environment ? and mental health of adolescents? Please put the following aspects in order of importance, putting the aspects that seem most important at the top. If you cannot assign some aspects, leave them unordered.**

current sex/ gender identity ? ☐

sex/ gender roles ? ☐

sexuality ? ☐

sex/ gender expression ? ☐

sex/ gender relations ? ☐

C2. If you wish, please comment on your rating!

C3. Do you know of any other gender concepts relevant for the social environment and mental health of adolescents?

No ☐

Yes [name of the concept and why important]: ☐

C4. How important do you find these gender approaches to adolescent mental health? Please rate each approach individually.

	very important	fairly important	important	slightly important	not at all important
Considering gender as a continuum between masculinity and femininity	☐	☐	☐	☐	☐
Multidimensionality approach ?	☐	☐	☐	☐	☐
Multi-level approach ?	☐	☐	☐	☐	☐
Intersectionality approach ?	☐	☐	☐	☐	☐
Gender power relations ?	☐	☐	☐	☐	☐
Embodiment approach ?	☐	☐	☐	☐	☐
Decolonial lens ?	☐	☐	☐	☐	☐

C5. If you wish, please comment on your rating!

C6.

No ☐

Yes [name of approach or theory and why important?]: ☐

C7.

C8. **Which gender norms do you think are of particular importance for the mental health of adolescents? Please write your ideas for gender norms and their possible impact for the mental health of adolescents into the text box. You can also repeat those you mentioned in the previous question.**

E.g. not sharing emotions -> can lead to substance misuse

C9. **Is there something you would like to add that has not been addressed in this part or is there something you would like to comment on?**

No ☐

Yes [please write a comment]: ☐

Section D: Section B: Mental health of adolescents

D1. **What aspects of adolescent mental health are particularly affected by gender norms? Please rate the importance of the following outcomes for adolescents' mental health according to how much they are affected by gender norms.**

	very important	fairly important	important	slightly important	not at all important
Mental, social and physical well-being	☐	☐	☐	☐	☐
Depressiveness	☐	☐	☐	☐	☐
Connectedness	☐	☐	☐	☐	☐
Body image	☐	☐	☐	☐	☐
Self-efficacy	☐	☐	☐	☐	☐
Coping	☐	☐	☐	☐	☐
Self-control	☐	☐	☐	☐	☐
Sense of coherence	☐	☐	☐	☐	☐
Happiness	☐	☐	☐	☐	☐
Life purpose	☐	☐	☐	☐	☐
Suicidal behaviour	☐	☐	☐	☐	☐
Resilience	☐	☐	☐	☐	☐
Risky behaviour	☐	☐	☐	☐	☐
Substance misuse	☐	☐	☐	☐	☐

D2. **If you wish, please comment on your rating!**

D3. **Do you know of any other mental health outcomes that are linked to experiences of gender for adolescents?**

No ☐

Yes [name of the mental health outcome(s) and why important?]: ☐

D4. **Is there something you would like to add that has not been addressed in this part or is there something you would like to comment on?**

No ☐

Yes [please write a comment]: ☐

Section E: Section C: Social environment of adolescents

E1. **We propose these five social environmental <u>levels</u> as particularly important for the gender norms of adolescents. Do you know of any other social environment levels relevant for experiences of gender for adolescents that are not portrayed in this illustration?**

?

No ☐

Yes [social environment level and why important?]: ☐

E2. **If you wish, please comment on the proposed social environment levels!**

E3. **In the different proposed social environment levels in the previous question, different actors may play a role for shaping the gender norms of adolescents. Which of the following actors are important to be included in the afore-mentioned levels? Please choose the level in which they play the greatest role.**

	individual level	interrelational level	community level	national level	global level	not relevant
School environment (teachers, classmates etc.)	☐	☐	☐	☐	☐	☐
Media (e.g. television, newspapers, movies etc.)	☐	☐	☐	☐	☐	☐
Social media (e.g. Tik-Tok, Instagram, Whats-app etc.)	☐	☐	☐	☐	☐	☐
Peers	☐	☐	☐	☐	☐	☐
Family	☐	☐	☐	☐	☐	☐
Sport groups	☐	☐	☐	☐	☐	☐
Faith groups	☐	☐	☐	☐	☐	☐
Healthcare providers	☐	☐	☐	☐	☐	☐
Political parties	☐	☐	☐	☐	☐	☐
Law enforcement	☐	☐	☐	☐	☐	☐
Civil society	☐	☐	☐	☐	☐	☐
Non-Profit Organisations	☐	☐	☐	☐	☐	☐

LimeSurvey

E4. **If you wish, please comment on your rating!**

E5. **Do you know of any other actors, groups or stakeholders that influence experiences of gender for adolescents?**

No ☐

Yes [name of actor and the social environment level and why important?]: ☐

E6. **What competencies are necessary for adolescents to navigate gender norms or expectations of their social environment in a way that allows them to develop in a psychologically healthy way?**

	very important	fairly important	important	slightly important	not at all important
Coping skills ?	☐	☐	☐	☐	☐
Agency ?	☐	☐	☐	☐	☐
Navigation ?	☐	☐	☐	☐	☐
Interpersonal relationship skills ?	☐	☐	☐	☐	☐
Critical reflection skills ?	☐	☐	☐	☐	☐
Mental health literacy ?	☐	☐	☐	☐	☐
Respect and empathy for others	☐	☐	☐	☐	☐

E7. **If you wish, please comment on your rating!**

LimeSurvey

E8. **Do you know of any other relevant competencies of adolescents that influence the way they navigate through the expectations of their social environment?**

No ☐

Yes [name of the competence and why important?]: ☐

E9. **Is there something you would like to add that has not been addressed in this part or is there something else you would like to comment on?**

No ☐

Yes [please write a comment]: ☐

Section F: Section D: Questions about your person

Thank you very much for sharing your knowledge and ideas with us!

The procedure is as follows: You will receive a PDF sheet with the feedback on the answers of all experts. This gives you the opportunity to check whether your opinion has been correctly and comprehensively reflected and whether you agree with the results of Delphi Round 1. This will be followed by Delphi Round 2.

F1. **In what year were you born?**

F2. **In which country did you acquire the most working experience?**

F3. **In which country do you live currently?**

LimeSurvey

F4. In which professional environment do you mainly operate?

Research ☐

Comment

[ ]

Development, services and implementation ☐

Comment

[ ]

Policy ☐

Comment

[ ]

Other ☐

Comment

[ ]

F5. Please specify in which professional environment you primarily evolve.

[ ]

F6. How confident are you in these areas?

	completely confident	fairly confident	somewhat confident	slightly confident	not confident at all	does not apply
Gender	☐	☐	☐	☐	☐	☐
Gender approaches, theories or concepts	☐	☐	☐	☐	☐	☐
Gender (norms) influencing adolescents	☐	☐	☐	☐	☐	☐
Gender socialisation of adolescents	☐	☐	☐	☐	☐	☐
Intersectionality (how gender intersects with other categories and structures)	☐	☐	☐	☐	☐	☐
Mental health	☐	☐	☐	☐	☐	☐
Mental health of adolescents	☐	☐	☐	☐	☐	☐
Other	☐	☐	☐	☐	☐	☐

LimeSurvey

F7. **Please specify this other area.**

F8. **What sex were you assigned at birth?**

- Male ☐
- Female ☐
- Intersex ☐

F9. **What is your current sex/ gender identity? We ask about gender identity and sexual orientation in this and the following question because it is best practice for surveys and is important for this research topic.**

- Female/ woman ☐
- Male/ man ☐
- Trans*/ transman / transwoman ☐
- Inter* ☐
- Non-binary ☐
- Queer ☐
- An identity not mentioned here ☐
- I do not want to classify as any sex/ gender category ☐

F10. **What is your sexual orientation?**

- Heterosexual ☐
- Lesbian or Gay ☐
- Bisexual ☐
- Or please specify: ☐
- Prefer not to say ☐

F11. **Is there anything else you would like to share with us?**

F12. **Please enter your e-mail address here so that we can send you the results of this Delphi round and contact you for the next round.**

Thank you for your participation in this first of three rounds of the Delphi survey! We very much appreciate your contributions. After the evaluation of this first round, you will receive a PDF sheet with the (anonymous) feedback on the answers of all experts.Thank you!
