# Supplementary material for: Correction: Gender and mental health of adolescents: A conceptual framework developed in a Delphi study
Source: PLoS One. 2026 Apr 8;21(4):e0346634. doi: 10.1371/journal.pone.0346634 (PMC13061248; doi:10.1371/journal.pone.0346634)
Supplement: S1 Data — (ZIP) [file pone.0346634.s001.zip › Limesurvey_Questionnaire Delphi round 2.pdf]

LimeSurvey

Thank you very much for your contributions in the first round of the Delphi study!

This questionnaire for the second round of the Delphi survey contains three sections. Section A is on gender (norms), section B on the mental health and section C on the social environment of adolescents.

How to fill out this questionnaire?

Your thoughts/ opinions on various theories, models or frameworks will be inquired. You might not be an expert on all topics, however, to combine these three constructs, it is necessary to gain new knowledge on the relations between these topics. Therefore, please try to fill out all sections from your perspective but always keep in mind that the questions are asked for and related to all three topics in this framework. There is no right or wrong to the questions so please feel free to fill out the questions according to your opinion or knowledge. You may feel like all proposed aspects are somehow important. The goal of this questionnaire is to reduce the aspects to the most relevant ones to ultimately develop the conceptual framework which can be used in quantitative research. If you do not know some terms, you can click on the green question mark to get more information. Please note that these are examples of a definition and that other meanings of the term are also possible.

--

Declaration of participation

For more information on the declaration of participation, please click on the term privacy policy or legal notice at the end of this page.

LimeSurvey

Section A: Pseudonymization

A1. **To identify you over the three Delphi rounds, we introduced a 4-digit acronym. Please enter the first two letters of your <u>mother's</u> name plus the two last numbers of <u>your</u> year of birth.**

E. g. Emma born in 1961 = EM61

Section B: Section A: Gender

B1. **These gender concepts are included in the conceptual framework:**

Gender identity ?

Sex assigned at birth ?

If any, which of those following gender concepts should also be included in the conceptual framework on gender (norms), the social environment and the mental health of adolescents? Please keep in mind that we can only include the most relevant aspects into the quantitative framework.

- Sex/gender expression ? ☐
- Sex/gender relations ? ☐
- Sex/gender roles ? ☐
- Sexual orientation (sexuality) ? ☐

B2. **If you wish, please comment on your rating!**

B3. **Are you aware of any (validated) instrument(s) that covers gender identity?**

- No ☐
- Yes ☐

B4. **Please tell us the name of the (validated) instrument(s).**

B5. **Please suggest possible questionnaire items that cover gender identity.**

B6. **Are you aware of any (validated) instrument(s) that covers sex assigned at birth?**

No ☐

Yes ☐

B7. **Please tell us the name of the (validated) instrument(s).**

B8. **Please suggest possible questionnaire items that cover sex assigned at birth.**

B9. **Are you aware of any (validated) instrument(s) that covers sex/gender expression?**

No ☐

Yes ☐

B10. **Please tell us the name of the (validated) instrument(s).**

B11. **Please suggest possible questionnaire items that cover sex/gender expression.**

B12. **Are you aware of any (validated) instrument(s) that covers sex/gender relations?**

No ☐

Yes ☐

B13. **Please tell us the name of the (validated) instrument(s).**

B14. **Please suggest possible questionnaire items that cover sex/gender relations.**

B15. **Are you aware of any (validated) instrument(s) that covers sex/gender roles?**

No ☐

Yes ☐

B16. **Please tell us the name of the (validated) instrument(s).**

B17. **Please suggest possible questionnaire items that cover sex/gender roles.**

B18. **Are you aware of any (validated) instrument(s) that covers sexual orientation (sexuality)?**

No ☐

Yes ☐

B19. **Please tell us the name of the (validated) instrument(s).**

B20. **Please suggest possible questionnaire items that cover sexual orientation (sexuality).**

B21. **These gender approaches are reflected in the conceptual framework:**

Multidimensionality approach ? Multilevel approach ? Intersectionality approach ? Gender power relations lens ?

If any, which of the following gender approaches should also be reflected in the conceptual framework? Please keep in mind that we can only include the most relevant aspects into the quantitative framework.

- Gender continuum ? ☐
- Gender spectrum ☐
- Embodiment approach ? ☐
- Decolonial lens ? ☐

B22. **If you wish, please comment on your rating!**

B23. **The gender approaches will not be presented as their own categories in the conceptual framework. Instead, they will be reflected by the other categories (gender, gender norms, social environment levels, mental health) and the way these categories are represented in the framework.**

For example: the multilevel approach is reflected in the different social environment levels that are relevant for adolescents and contain actors as carriers of gender norms.

How can the multidimensionality approach be reflected in the framework?

B24. **How can the multilevel approach be reflected in the framework?**

B25. **How can the intersectionality approach be reflected in the framework?**

B26. **How can the power relations lens be reflected in the framework?**

B27. **How can the gender continuum be reflected in the framework?**

B28. **How can the gender spectrum be reflected in the framework?**

B29. **How can the embodiment approach be reflected in the framework?**

B30. **How can the decolonial lens be reflected in the framework?**

B31.

Derived from all your proposed gender norms, we are suggesting categorizations for gender norms. We are suggesting two axes of categorizations.

1: This is our *first* suggestion for a categorization of gender norms (for boys and girls). It was derived from the suggestions in round 1 and covers the content-related areas where gender norms occur.

Behaviour norms ? Body and appearance norms ? Sexual and relationship norms ? Performance norms ? Education norms ? Mobility norms ? Career norms ?

2: This is our *second* suggestion for a categorization of gender norms. This suggestion adds descriptive ? and prescriptive norms ? to the content-related areas of gender norms.

Descriptive norms Prescriptive norms Behaviour norms E.g.:
Boys *are* stoic
Girls *are* emotional E.g.:
Boys *should* be strongGirls *should* be empathic Body and appearance norms Sexual and relationship norms Performing norms Education norms Mobility norms Career norms

Suggestion 1 ☐

Suggestion 2 ☐

None of these suggestions ☐

LimeSurvey

B32. **If you wish, please comment on your rating!**

B33. **If you want, please propose another suggestion for a categorization of gender norms.**

B34. **Are you aware of any existing models/theories/frameworks that include any relevant categorization of gender norms? If so, please describe.**

B35. **Is there something you would like to add that has not been addressed in this part or is there something you would like to comment on?**

No ☐

Yes [please write a comment]: ☐

Section C: Section B: Mental health

C1. **These mental health outcomes are included in the conceptual framework:**

Mental, social and physical well-being Depressiveness Connectedness Body image Happiness Risky behaviour

If any, which of the following mental health outcomes should also be included in the conceptual framework on gender (norms), the social environment and the mental health of adolescents? Please keep in mind that we can only include the most relevant aspects into the quantitative framework.

- Self-efficacy ☐
- Self-control ☐
- Life purpose ☐
- Suicidal behaviour ☐
- Resilience ☐
- Substance misuse ☐
- Sense of coherence ☐

C2. **If you wish, please comment on your rating!**

C3. **Are you aware of any (validated) instrument(s) that covers mental, social and physical well-being?**

- No ☐
- Yes ☐

C4. **Please tell us the name of the (validated) instrument(s).**

LimeSurvey

C5. **Please suggest possible questionnaire items that cover mental, social and physical well-being.**

C6. **Are you aware of any (validated) instrument(s) that covers depressiveness?**

No ☐

Yes ☐

C7. **Please tell us the name of the (validated) instrument(s).**

C8. **Please suggest possible questionnaire items that cover depressiveness.**

C9. **Are you aware of any (validated) instrument(s) that covers connectedness?**

No ☐

Yes ☐

C10. **Please tell us the name of the (validated) instrument(s).**

C11. **Please suggest possible questionnaire items that cover connectedness.**

C12. **Are you aware of any (validated) instrument(s) that covers body image?**

No ☐

Yes ☐

C13. **Please tell us the name of the (validated) instrument(s).**

C14. **Please suggest possible questionnaire items that cover body image.**

C15. **Are you aware of any (validated) instrument(s) that covers happiness?**

No ☐

Yes ☐

C16. **Please tell us the name of the (validated) instrument(s).**

C17. Please suggest possible questionnaire items that cover happiness.

C18. Are you aware of any (validated) instrument(s) that covers risky behaviour?

No ☐

Yes ☐

C19. Please tell us the name of the (validated) instrument(s).

C20. Please suggest possible questionnaire items that cover risky behaviour.

C21. Are you aware of any (validated) instrument(s) that covers self-efficacy?

No ☐

Yes ☐

C22. Please tell us the name of the (validated) instrument(s).

C23. **Please suggest possible questionnaire items that cover self-efficacy.**

C24. **Are you aware of any (validated) instrument(s) that covers self-control?**

No ☐

Yes ☐

C25. **Please tell us the name of the (validated) instrument(s).**

C26. **Please suggest possible questionnaire items that cover self-control.**

C27. **Are you aware of any (validated) instrument(s) that covers life purpose?**

No ☐

Yes ☐

C28. **Please tell us the name of the (validated) instrument(s).**

LimeSurvey

C29. Please suggest possible questionnaire items that cover life purpose.

C30. Are you aware of any (validated) instrument(s) that covers suicidal behaviour?

No

Yes

C31. Please tell us the name of the (validated) instrument(s).

C32. Please suggest possible questionnaire items that cover suicidal behaviour.

C33. Are you aware of any (validated) instrument(s) that covers resilience?

No

Yes

C34. Please tell us the name of the (validated) instrument(s).

C35. Please suggest possible questionnaire items that cover resilience.

C36. Are you aware of any (validated) instrument(s) that covers substance misuse?

No

Yes

C37. Please tell us the name of the (validated) instrument(s).

C38. Please suggest possible questionnaire items that cover substance misuse.

C39. Are you aware of any (validated) instrument(s) that covers sense of coherence?

No

Yes

C40. Please tell us the name of the (validated) instrument(s).

C41. Please suggest possible questionnaire items that cover sense of coherence.

C42. Is there something you would like to add that has not been addressed in this part or is there something else you would like to comment on?

Section D: Section C: Social environment of adolescents

The following illustration of social environment levels was updated based on the suggestions in the previous Delphi round.

Please note that the social environment levels are not operationalised themselves. The levels serve as “buckets” for the actors who are carriers of gender norms. Please keep in mind that we can only include the most relevant aspects into the quantitative framework.

D1. Do you think this illustration is appropriate for the conceptual framework?

No ☐

Yes ☐

D2. Please make another suggestion for the social environment levels.

D3. **If you wish, please comment on your rating!**

D4. **The following competencies for adolescents are included in the conceptual framework:**

Coping skills ? Agency ? Interpersonal relationship skills ? Critical reflection skills ? Mental health literacy ? Respect and empathy for others

If any, which of those competencies for adolescents should also be included in the conceptual framework on gender (norms), the social environment and the mental health of adolescents? Please keep in mind that we can only include the most relevant aspects into the quantitative framework.

- Media literacy ☐
- Self-awareness ☐
- Life literacy ? ☐
- Self-efficacy ☐
- Spirituality ☐
- Assertive skills ☐
- Navigation ? ☐

D5. **If you wish, please comment on your rating!**

D6. **Are you aware of any (validated) instrument(s) that covers coping skills?**

- No ☐
- Yes ☐

LimeSurvey

D7. **Please tell us the name of the (validated) instrument(s).**

D8. **Please suggest possible questionnaire items that cover coping skills.**

D9. **Are you aware of any (validated) instrument(s) that covers agency?**

No ☐

Yes ☐

D10. **Please tell us the name of the (validated) instrument(s).**

D11. **Please suggest possible questionnaire items that cover agency.**

D12. **Are you aware of any (validated) instrument(s) that covers interpersonal relationship skills?**

No ☐

Yes ☐

D13. **Please tell us the name of the (validated) instrument(s).**

D14. **Please suggest possible questionnaire items that cover interpersonal relationship skills.**

D15. **Are you aware of any (validated) instrument(s) that covers critical reflection skills?**

No ☐

Yes ☐

D16. **Please tell us the name of the (validated) instrument(s).**

D17. **Please suggest possible questionnaire items that cover critical reflection skills.**

D18. **Are you aware of any (validated) instrument(s) that covers mental health literacy?**

No ☐

Yes ☐

LimeSurvey

D19. **Please tell us the name of the (validated) instrument(s).**

D20. **Please suggest possible questionnaire items that cover mental health literacy.**

D21. **Are you aware of any (validated) instrument(s) that covers respect and empathy for others?**

No ☐

Yes ☐

D22. **Please tell us the name of the (validated) instrument(s).**

D23. **Please suggest possible questionnaire items that cover respect and empathy for others.**

D24. **Are you aware of any (validated) instrument(s) that covers media literacy?**

No ☐

Yes ☐

D25. **Please tell us the name of the (validated) instrument(s).**

D26. **Please suggest possible questionnaire items that cover media literacy.**

D27. **Are you aware of any (validated) instrument(s) that covers self-awareness?**

No ☐

Yes ☐

D28. **Please tell us the name of the (validated) instrument(s).**

D29. **Please suggest possible questionnaire items that cover self-awareness.**

D30. **Are you aware of any (validated) instrument(s) that covers life literacy?**

No ☐

Yes ☐

D31. **Please tell us the name of the (validated) instrument(s).**

D32. **Please suggest possible questionnaire items that cover life literacy.**

D33. **Are you aware of any (validated) instrument(s) that covers self-efficacy?**

No ☐

Yes ☐

D34. **Please tell us the name of the (validated) instrument(s).**

D35. **Please suggest possible questionnaire items that cover self-efficacy.**

D36. **Are you aware of any (validated) instrument(s) that covers spirituality?**

No ☐

Yes ☐

D37. **Please tell us the name of the (validated) instrument(s).**

D38. **Please suggest possible questionnaire items that cover spirituality.**

D39. **Are you aware of any (validated) instrument(s) that covers assertive skills?**

No ☐

Yes ☐

D40. **Please tell us the name of the (validated) instrument(s).**

D41. **Please suggest possible questionnaire items that cover assertive skills.**

D42. **Are you aware of any (validated) instrument(s) that covers navigation?**

No ☐

Yes ☐

D43. **Please tell us the name of the (validated) instrument(s).**

D44. **Please suggest possible questionnaire items that cover navigation.**

D45. **Is there something you would like to add that has not been addressed in this part or is there something else you would like to comment on?**

No ☐

Yes [please write a comment]: ☐

D46. **Is there anything else you would like to share with us?**

Thank you for your participation in this second of three rounds of the Delphi survey! We very much appreciate your contributions.

After the evaluation of this second round, you will receive a PDF sheet with the (anonymous) feedback on the answers of all experts.Thank you!
