# Supplementary material for: Correction: Gender and mental health of adolescents: A conceptual framework developed in a Delphi study
Source: PLoS One. 2026 Apr 8;21(4):e0346634. doi: 10.1371/journal.pone.0346634 (PMC13061248; doi:10.1371/journal.pone.0346634)
Supplement: S1 Data — (ZIP) [file pone.0346634.s001.zip › Limesurvey_Questionnaire Delphi round 3.pdf]

Thank you very much for your contributions in the second round of the Delphi study!

This questionnaire for the third and final round of the Delphi survey is the shortest of all rounds and takes approximately 10-15 minutes to complete.

How to fill out this questionnaire?

Your thoughts/ opinions on various theories, models or frameworks will be inquired. You might not be an expert on all topics, however, to combine these three constructs, it is necessary to gain new knowledge on the relations between these topics. Therefore, please try to fill out all sections from your perspective but always keep in mind that the questions are asked for and related to all three topics in this framework. There is no right or wrong to the questions so please feel free to fill out the questions according to your opinion or knowledge. You may feel like all proposed aspects are somehow important. The goal of this questionnaire is to reduce the aspects to the most relevant ones to ultimately develop the conceptual framework which can be used in quantitative research.

--

Declaration of participation

For more information on the declaration of participation, please click on the term privacy policy or legal notice at the end of this page.

Section A: Pseudonymization

A1. **To identify you over the three Delphi rounds, we introduced a 4-digit acronym. Please enter the first two letters of your mother's name plus the two last numbers of your year of birth.**

E. g. Emma born in 1961 = EM61

Section B: Section A

These are the constructs for the conceptual framework that are based on the results of Delphi round 2.

This initial conceptual framework includes the constructs that reached consensus in the first two Delphi rounds: Gender norms of social environment, sex assigned at birth, gender identity, gender norms, competencies, gender roles and mental health.

Gender norms of social environment are measured by gender norms of (actors of) the household level; gender norms of (actors of) the community level; gender norms of (actors of) the political level and gender norms of (actors of) the digital level. (Individual) gender norms are measured by behaviour norms; performance norms; body & appearance norms; education norms; sexual & relationship norms; career norms and mobility norms. Competencies are measured by coping skills; agency skills; respect & empathy; mental health literacy; critical reflection skills and interpersonal relationship skills. Mental health, depending on the research interest, is measured either by mental, social or physical well-being; depressiveness; connectedness; body image; resilience; happiness or risky behaviour.

We further integrated several gender approaches either directly or indirectly.

We integrated the multilevel approach by integrating several social environment levels. Additionally, we reflected the multidimensionality approach by developing sex and gender concepts with different dimensions (sex assigned at birth, gender identity, gender roles). We reflected the intersectionality approach and the gender power relations lens by integrating multiple social categories/intersectional variables for the population group of adolescents that include aspects of power relations and processes of discrimination and touch upon the micro level but also reflect multiple interlocking systems of privilege and oppression at the macro level.

We are now looking for assumptions about causal relationships (one event causes the effect of another event, also referred to as cause-and-effect relationships) between these constructs for possible quantitative analyses.

B1. **If you wish, please comment on the statements above!**

B2. **Starting from sex assigned at birth, with which other construct(s) (in blue) would you assume a causal relationship?**

Sex assigned at birth has an influence on individual gender norms ☐

Sex assigned at birth has an influence on gender roles ☐

Sex assigned at birth has an influence on competencies ☐

Sex assigned at birth has an influence on mental health ☐

Sex assigned at birth has an influence on gender identity ☐

Sex assigned at birth has an influence on gender norms of social environment ☐

B3. Starting from current gender identity, with which other construct(s) (in blue) would you assume a causal relationship?

Gender identity has an influence on gender norms ☐

Gender identity has an influence on gender roles ☐

Gender identity has an influence on competencies ☐

Gender identity has an influence on mental health ☐

Gender identity has an influence on gender norms of social environment ☐

B4. Starting from gender norms of the social environment, with which other construct(s) (in blue) would you assume a causal relationship?

Gender norms of social environment have an influence on gender norms ☐

Gender norms of social environment have an influence on gender roles ☐

Gender norms of social environment have an influence on competencies ☐

Gender norms of social environment have an influence on mental health ☐

Gender norms of social environment have an influence on gender identity ☐

B5. Starting from (individual) gender norms, with which other construct(s) (in blue) would you assume a causal relationship?

Gender norms have an influence on gender roles ☐

Gender norms have an influence on competencies ☐

Gender norms have an influence on mental health ☐

Gender norms have an influence on gender identity ☐

Gender norms have an influence on gender norms of social environment ☐

B6. Starting from gender roles, with which other construct(s) (in blue) would you assume a causal relationship?

Gender roles have an influence on gender norms ☐

Gender roles have an influence on competencies ☐

Gender roles have an influence on mental health ☐

Gender roles have an influence on gender identity ☐

Gender roles have an influence on gender norms of social environment ☐

B7. **Starting from competencies, with which other construct(s) (in blue) would you assume a causal relationship?**

Competencies have an influence on gender norms ☐

Competencies have an influence on gender roles ☐

Competencies have an influence on mental health ☐

Competencies have an influence on gender identity ☐

Competencies have an influence on gender norms of social environment ☐

B8. **If you wish, please comment on your ratings!**

Section C: Section B

C1.

In the last Delphi round, there was a consensus on including gender roles in the conceptual framework. We propose operationalising gender roles with "time spent on gender-typed activities" (e.g. domestic chores for household, work in family business/income generating activities, care for others).

Do you find this proposition adequate to operationalise gender roles of adolescents?

Yes ☐

No ☐

C2. **Please suggest another operationalisation for gender roles.**

C3. **Please take a look at the suggested social positions forming an intersectional perspective (in light green on the left of the proposed conceptual framework). Do you find the proposed social positions relevant for adolescents?**

Yes ☐

No ☐

C4. **Please make another suggestion for the social position variables.**

C5. **Do you find the proposed social positions complete for an intersectional perspective?**

Yes ☐

No ☐

C6. **Please make another suggestion for the social position variables.**

C7. **In the last Delphi round, we concluded on the social environment levels (individual, household, community, political, digital). Please select the actors who are most likely to influence adolescents' gender norms. We integrated your suggestions from the first Delphi round.**

Family ☐

Peers/Friends ☐

School environment [e.g. teachers, classmates etc.] ☐

Sport group ☐

Faith club ☐

Clubs (hobby-based groubs) ☐

Role models ☐

Celebrities ☐

Influencers ☐

Traditional leaders ☐

Media [e.g. television, newspapers, movies etc.] ☐

Social Media [e.g. Tik-Tok, Instagram etc.] ☐

Pornography ☐

Workplace ☐

Healthcare providers ☐

Political parties ☐

Law enforcement ☐

Civil Society ☐

Non-Profit Organisations ☐

C8. Please assign the actor family to the social environment level in which it plays the greatest role.

Household level ☐

Community level ☐

Political (includes institutional) level ☐

Digital level ☐

C9. Please assign the actor peers/friends to the social environment level in which it plays the greatest role.

Household level ☐

Community level ☐

Political (includes institutional) level ☐

Digital level ☐

C10. Please assign the actor school environment to the social environment level in which it plays the greatest role.

Household level ☐

Community level ☐

Political (includes institutional) level ☐

Digital level ☐

C11. Please assign the actor sport group to the social environment level in which it plays the greatest role.

Household level ☐

Community level ☐

Political (includes institutional) level ☐

Digital level ☐

C12. Please assign the actor faith group to the social environment level in which it plays the greatest role.

Household level ☐

Community level ☐

Political (includes institutional) level ☐

Digital level ☐

C13. Please assign the actor hobby-based clubs to the social environment level in which it plays the greatest role.

- Household level ☐
- Community level ☐
- Political (includes institutional) level ☐
- Digital level ☐

C14. Please assign the actor role models to the social environment level in which it plays the greatest role.

- Household level ☐
- Community level ☐
- Political (includes institutional) level ☐
- Digital level ☐

C15. Please assign the actor celebrities to the social environment level in which it plays the greatest role.

- Household level ☐
- Community level ☐
- Political (includes institutional) level ☐
- Digital level ☐

C16. Please assign the actor influencers to the social environment level in which it plays the greatest role.

- Household level ☐
- Community level ☐
- Political (includes institutional) level ☐
- Digital level ☐

C17. Please assign the actor traditional leaders to the social environment level in which it plays the greatest role.

- Household level ☐
- Community level ☐
- Political (includes institutional) level ☐
- Digital level ☐

LimeSurvey

C18. **Please assign the actor media to the social environment level in which it plays the greatest role.**

- Household level ☐
- Community level ☐
- Political (includes institutional) level ☐
- Digital level ☐

C19. **Please assign the actor social media to the social environment level in which it plays the greatest role.**

- Household level ☐
- Community level ☐
- Political (includes institutional) level ☐
- Digital level ☐

C20. **Please assign the actor pornography to the social environment level in which it plays the greatest role.**

- Household level ☐
- Community level ☐
- Political (includes institutional) level ☐
- Digital level ☐

C21. **Please assign the actor workplace to the social environment level in which it plays the greatest role.**

- Household level ☐
- Community level ☐
- Political (includes institutional) level ☐
- Digital level ☐

C22. **Please assign the actor healthcare providers to the social environment level in which it plays the greatest role.**

- Household level ☐
- Community level ☐
- Political (includes institutional) level ☐
- Digital level ☐

LimeSurvey

C23. Please assign the actor political parties to the social environment level in which it plays the greatest role.

- ☐ Household level
- ☐ Community level
- ☐ Political (includes institutional) level
- ☐ Digital level

C24. Please assign the actor law enforcement to the social environment level in which it plays the greatest role.

- ☐ Household level
- ☐ Community level
- ☐ Political (includes institutional) level
- ☐ Digital level

C25. Please assign the actor Civil Society to the social environment level in which it plays the greatest role.

- ☐ Household level
- ☐ Community level
- ☐ Political (includes institutional) level
- ☐ Digital level

C26. Please assign the actor Non-Profit Organisations to the social environment level in which it plays the greatest role.

- ☐ Household level
- ☐ Community level
- ☐ Political (includes institutional) level
- ☐ Digital level

C27. If you wish, please comment on your ratings!

C28. **Is there anything you would like to mention or ameliorate concerning the conceptual framework at this stage?**

C29. **Looking back at the three Delphi rounds. Is there anything you would like to share with us?**

Thank you for your participation in the third and final round of the Delphi survey! We very much appreciate your contributions and your time and effort over the three rounds!

After the evaluation of this last round, you will receive a PDF sheet with the (anonymous) feedback on the answers of all experts. We will also share with you the final conceptual framework. Thank you!
